# Correction: Comparative analysis of the Potter Tower and a new Track Sprayer for the application of residual sprays in the laboratory

**DOI:** 10.1186/s13071-026-07272-w

**Published:** 2026-03-07

**Authors:** Jane Bonds, George Parsons, Kyle J. Walker, Annabel Murphy, Rosemary Susan Lees, Derric Nimmo, John Clayton, David Malone

**Affiliations:** 1 Bonds Consulting Group LLC, 3900 Wasp Street, Panama City Beach, FL 32408 USA; 2 Department of Vector Biology, Liverpool School of Tropical Medicine, Liverpool, L3 5QA UK; 3 Innovation to Impact, Liverpool School of Tropical Medicine, Pembroke Place, Liverpool, L3 5QA UK; 4 Innovative Vector Control Consortium (IVCC), Liverpool School of Tropical Medicine, Liverpool, L3 5QA UK; 5 Micron Sprayers Ltd, Bromyard Industrial Estate, Bromyard, HR7 4HS UK; 6 Bill & Melinda Gates Foundation, 500 5th Ave N, Seattle, WA 98109 USA

**Correction: Parasites & Vectors (2024) 17:66** 10.1186/s13071-024-06168-x

Following publication of the original article [1], the authors flagged the following concern regarding how the data on variability of spray dose was analyzed:

"Where the original analysis measured average spray dose and variation in spray dose for a sample of 4 of the cover slips sprayed with the Track Sprayer, to match the number of cover slips sprayed by the Potter Tower, we have repeated the analysis to include all 9 cover slips for the TS, which we have realized is better practice. This change increases the coefficient of variance for the track sprayer, and increases from 3 to 4 the number of cover slips across the 3 runs which were outside the 10% pass/fail metric, but does not change the conclusions of the paper."

In addition, they flagged that the corresponding author email was incorrect, that the availability of data and materials declaration was incomplete (it did not provide a link to raw data of the article), and several minor errors in formatting/wording.

The article has since been updated to correct the errors. For details of the exact corrections, please see Additional file 1 of this erratum article, which is a copy of the original article annotated with the post-publication corrections in question.

## Supplementary Information

Additional file 1.
