# Supplementary material for: Comparative analysis of the Potter Tower and a new Track Sprayer for the application of residual sprays in the laboratory
Source: Parasit Vectors. 2024 Feb 16;17:66. doi: 10.1186/s13071-024-06168-x (PMC10870534; doi:10.1186/s13071-024-06168-x)
Supplement: Supplementary file 1 — Additional file 1. Standard Operating Procedure (SOP): Application of compounds to surfaces using the Potter Tower, provided by the Liverpool Insect Testing Establishment (LITE) at the Liverpool School of Tropical Medicine (LSTM). [file 13071_2024_6168_MOESM1_ESM.pdf]

SOP: Spraying Insecticides with the Potter Tower

July 2022

**Title**	Spraying Insecticides with the Potter Tower
**Document number**	I2I-SOP-012
**Version number**	2
**Date first published**	30/10/2020
**Date last revised**	19/07/2022

Prepared by

Name	Role	Institution
Alex Wright	Author	Consultant to I2I
Graham Small	Author	IVCC
KCMUCo	Contributor	KCMUCo
Natalie Lissenden	Contributor	LSTM
Katherine Gleave	Contributor	LSTM

Timeline

Version	Date	Reviewed by	Institution
1	30/10/2020	Angus Spiers	I2I
2	19/07/2022	Angus Spiers<br>Rosemary Lees	I2I<br>LSTM

Version Control[1]

Version	Date	Updated by	Description of update(s)
2	June-July 2022	Alex Wright, Katherine Gleave	Related documents, purpose, materials & equipment, data collection sheet information, health and safety, glossary of terms and references added.

[1] Historical versions of SOPs can be found on the I2I website (https://innovationtoimpact.org/)

Related documents

- I2I Best Practice SOP Library, 30 October 2020 (https://innovationtoimpact.org/)
    - SOP- Cone bioassay of IRS Treated Blocks (I2I-SOP-010)
    - SOP- IRS Block Preparation (I2I-SOP-011)

1. Purpose

The Potter Tower is used to deliver insecticide droplets accurately onto blocks made of mud, concrete, plywood and tiles for subsequent assays in the test rooms and onto filter papers to verify spraying accuracy. The pressurized spray nozzle of the Potter Tower produces a consistent uniform spray onto the substrate positioned on the spray platform below the spray column. Liquids put in the delivery vial at the top are drawn through an atomiser by the suction force of a compressed air cylinder connected to it.

The potter tower is most suitable for insecticides that have been supplied in small volumes by the sponsors, as it is more efficient (less total volume is required to achieve the desired deposit on the substrates) than the spinning disk sprayer, which is the alternative method if the potter tower requires maintenance at any point.

2. Background

The World Health Organisation (WHO) cone is 12 cm in diameter, the minimum size required for a substrate sample to be tested. Blocks of cement, plaster or mud, 1-1.2 cm thick, are prepared in Petri dishes and dried at 27 °C ± 2 °C and 80% ± 10% Relative Humidity (RH). These and the substrates not requiring pre-preparation, such as wood or thatch, are sprayed with insecticide to make a homogeneous residual deposit of the desired concentration of active ingredient per unit area. Spraying is done using a Potter Spray Tower®, which is internationally recognized as the most precise method of chemical spraying in the laboratory. All substrate samples are then stored unsealed under controlled temperature conditions (30 °C ± 2 °C), humidity (80±10%RH), air circulation and ambient light cycles until ready for testing. A minimum of seven replicate

blocks per dosage are prepared, at least three for bioassay and four for initial chemical analysis, selected at random.

3. Materials and equipment

3.1. Centralizing the nozzle arms and calibration.

- Four glass cover slips
- Weigh scale
- Control solution (50% glycerol)
- Data sheets

3.2. Preparation before first collection

- 70% ethanol solution
- Paper towels
- Biohazard bags

3.3. Spraying

- Half mask aspirator
- Nitrile gloves
- Lab coat
- Goggles and visor
- Spray suit
- Over boots
- Pipettes
- Filtered pipette tips
- Mixing spoons
- Deionized water
- Glass beakers
- Glass cylinders

- Weigh boats
- Marker pens
- Insecticide (test item)
- Marker pen
- Labels
- Aluminum foil
- Plastic bags

3.4. Cleaning and disposal

- Jerry can
- Biohazard bags
- Soap solution
- 70% ethanol and spray bottle

4. Procedure

4.1. Levelling the tower and nozzle arms

4.1.1. It is important that both the tower and the nozzle are completely level otherwise the spray will be delivered imperfectly and unevenly onto the blocks.

4.1.2. Check that the tower is level by checking that the spirit level bubble of the tower is inside the circle. The spirit level is located on the tower itself at the front. Adjust the feet of the tower at the base until the bubble is inside the circle. Record that this has been done and record if any adjustments were made.

4.1.3. Next check that the spray nozzle arms are level. Place the spirit level (located in the cupboard in the outer area of the Spray Room) across the arms in turn (i.e. arms A&B, B&C, C&A) and adjust the screws until the arms are all level with each other. Record that this has been done, and record if any adjustments were made.

4.2. Centralizing the nozzle arms.

4.2.1. The nozzle must also be central within the Spray Column or the spray will not be delivered vertically downwards or evenly across the block.

4.2.2. Weigh 4 glass coverslips on the weigh scale. Check the weigh scale is level, and do not use any weigh scales that do not weigh to 4 decimal places. Record the weight of each coverslip next to the letters A-D on the form. Do not label the coverslips A-D but remember where the coverslips have been placed and do not mix them up.

4.2.3. Place the centralising bungs apparatus (the bungs are glued to a petri dish to stop them moving - they prevent the coverslips absorbing water from the spray platform) onto the spray platform and arrange the coverslips onto the apparatus, using the pins to hold them in place, to form a square in the centre with A-D arranged correctly. Hold the coverslips by the sides only:

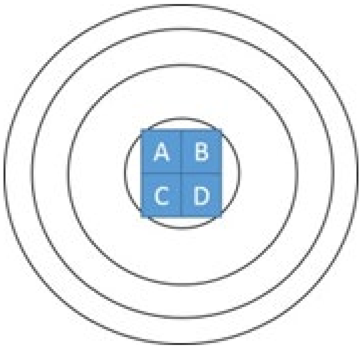

4.2.4. Put 2mL of the 50% glycerol solution into the Potter Tower delivery vial (use the vial labelled CONTROL) and spray it.

4.2.5. Remove the coverslips from the bungs and re-weigh the coverslips, recording the value next to the correct position of each coverslip (A-D) on the form.

4.2.6. Calculate the difference between the initial weight and the weight after spraying and record those values.

4.2.7. If the difference for each coverslip (the amount deposited) varies by more than 5% of the mean of the four coverslips, make adjustments to the nozzle angle by turning the screws of the nozzle arms slightly. Then repeat steps 3.2.3 to 3.2.6 until values are within the acceptable range. Use multiple copies of your data entry form as needed if values are out of range. Staple all forms together that are part of a single Potter tower preparation process.

4.3. Cleaning before spraying

(NB. Before every use of the Potter Tower for spraying insecticides it should be cleaned, regardless of how recently it was used.)

4.3.1. Pipette 5mL of 70% ethanol solution into the delivery vial of the Potter Tower and spray it through the nozzle.

4.3.2. Wash down the walls of the Potter Tower spray column using 70% ethanol. Wipe the internal surfaces and spray platform of the potter tower with disposable paper towels. These must be disposed of in the biohazard bags as it could be contaminated by contact with the Potter Tower.

4.3.3. Repeat steps 3.3.1 and 3.3.2 with tap water only, 3 times (i.e. a total of 15mL in the delivery vial).

4.3.4. Record on the form that the cleaning has been done and record the technician initials.

4.4. Calibration of spray output with water.

Most of the content of the delivery vial is not delivered on to the block on the spray platform but coats the side of the spray column. Because of this it is necessary to know how much actually reaches the block and to adjust the calculations for the total volume of insecticide solution needed. This calibration of the spray output should be done with tap water unless otherwise indicated by the Study Director in the protocol (for very viscous test items it may be necessary to use other types of calibration).

4.4.1.Measure out a pre-determined volume of tap water using a pipette and add it into the delivery vial. As a guide, typically between 2-4mL is the right amount to be able to deliver the target weight on to a 9cm diameter block. For the same size block, the target spray weight is 0.254g with an acceptable range of 10% i.e. between 0.23-0.28g.

4.4.2. Having ensured that the balance has been correctly calibrated, weigh the untreated calibration block (of the same substrate that you are going to spray

with insecticides after calibration). If spraying multiple substrates use all relevant calibration blocks (i.e. perform multiple calibrations with water).

4.4.3. Tare the scales and place the block on the spray platform.

4.4.4. If necessary, adjust the circular clips that hold the block in place to position the block centrally on the spray platform.

4.4.5. The windows should always be closed in the Spray Room, but double check that they are before spraying, and turn off the A/C if it is on. The ventilator should continue running even during spraying.

4.4.6. Turn on the compressor, checking that the pressure is between 15-20psi. Operate the spray lever (the black on/off switch on the front of the Potter Tower). The spray platform should close automatically.

4.4.7. Once the delivery vial has emptied, turn off the Potter Tower at the spray lever (the spray platform will open automatically) then turn off the compressor. It is important to turn off the compressor between blocks otherwise it will overheat if it is left on for a long time.

4.4.8. Remove the block, holding it carefully by the sides. Do not touch the surface.

4.4.9. Immediately reweigh the block and record this value as the actual spray weight on the form.

4.4.10. If the weight is not within 10% range then the decision is “rejected”. If within range then “accepted”.

4.4.11. If the block is rejected then an adjustment must be made – if over sprayed then reduce the quantity put in the delivery vial, and vice versa. Wipe the spray platform with disposable paper towel before spraying a second time. The same calibration block can be reused. Continue to make small adjustments and repeat steps 3.4.1 to 3.4.10 until the target spray weight is achieved. All spray runs must be recorded regardless of whether they are accepted or rejected.

4.4.12. There must be three consecutive acceptable spray runs before you continue to the next step. Make sure you have recorded all information before proceeding.

4.5. Calculations

The calculations should be done once the calibration steps (section 3.4) are completed, as the delivery vial volume is needed as the adjustment value in the calculation of the total volume to be sprayed.

4.5.1. The date the block is made is written on the block label on the underside of each block. Usually a series of blocks all with the same preparation date (i.e. date block made) are chosen for one protocol. If the preparation dates differ but are within seven days of each other, this is an acceptable batch of blocks to use for the same protocol. In this case, record the earliest of the dates and note the range of dates down.

4.5.2. Write the insecticide code used on the recording form. If spraying multiple insecticide formulations on the same day, use a separate form for each separate formulation and/or AI.

4.5.3. The chemical code and lot number can be found on the container of the insecticide stock solution. You should fill these sections only after asking the Study Director, Project Manager or Facility Supervisor to get the insecticide(s) that you need for spraying from the Chemical Storeroom.

4.5.4. The number of blocks, filter papers and tiles can be found in the protocol. Ask the Study director, Project Manager or Lab Supervisor if this is not clear.

4.5.5. Typically blocks made in petri dishes of 8.5cm diameter are sprayed. The surface area of these blocks is 0.0056m2. If using a different size of block, filter paper or tile (filter papers and tiles are also usually cut to the same size) then you must calculate the surface area.

4.5.6. Record the target active ingredient concentration application rate and the application method which should all be stated in the protocol.

4.5.7. The amount of active ingredient (AI) in the stock solution should be written as mg per mL of inert liquid (if stock is a liquid, like an SC formulation) or as mg per g of inert material (if stock is a solid, like a WG or WP formulation). You must

write the correct unit depending on the state (solid/liquid) of the stock The AI amount should always be expressed in mg

4.5.8. Write the value for the amount of water if the stock is a solid then this is the same value as written for If it is a liquid then the amount of water to use should be the total volume minus the amount of stock solution liquid.

4.5.9. If the stock solution is a liquid and the amount required is less than 1mL you should use exactly 1mL of the stock solution and adjust the amount of water to use. This is because it is hard to measure out less than 1mL of the insecticide accurately, particularly if it is quite viscous.

4.5.10. If the stock solution is a liquid and the amount required is equal to or more than 1mL you do not need to adjust the stock to use or the water to mix with it. If the stock is solid and the amount required is less than 1mg you should use exactly 1mg and adjust the amount of water to mix with it. This is because it is hard to measure out less than 1mg of the insecticide accurately.

4.6. Weighing and mixing the insecticide stock and the water (or another dilutent)

4.6.1. Ask the Study Director, Project Manager or Facility Supervisor to get the insecticide(s) that you need for spraying from the Chemical Storeroom. They are the only staff authorised to access the Storeroom and they need to complete the Chemical Usage Record Sheet for each insecticide that you use.

4.6.2. Weighing and mixing the insecticides must only be done in the Spray Room. This should be done in the fume hood if it is in service, but it is important to remember that the fume hood is the secondary level of protection and full PPE is still the primary level of protection.

4.6.3. Before starting, put on your own half-mask respirator, nitrile gloves, lab coat and goggles as the minimum PPE required. This is for weighing only. Before you spray you must replace the lab coat with the spray suit and the goggles for a visor, plus put on the over boots. If you only have to make one or two insecticide solutions then you can put on full spray PPE as the weighing will not take much time.

4.6.4. Prepare all the materials first before opening the insecticide. This means having pipette(s), pipette tips (filtered), mixing spoons, deionised water, a tray for the insecticide to stand in, glass beakers, glass cylinders, weigh boats, marker pens, as well as the insecticide needed all ready and within reach.

4.6.5. Do not pipette directly from the insecticide stock bottle. Pour into a smaller beaker, or a weighing boat, and pipette the liquid from there. Shake the insecticide stock container well before measuring.

4.6.6. Measure the insecticide and the deionised water (always use deionised water for mixing with the insecticide unless specified in the protocol) using a pipette for volumes less than 10mL (i.e. if measuring 4mL use the 1mL pipette 4 times). Use a gradated measuring cylinder of appropriate size for volumes greater than 10mL. For example, if you need to measure 85mL of deionised water, use the 100mL cylinder, do not use the 1L cylinder.

4.6.7. Once measured, pour some of the deionised water into a mixing beaker of appropriate size. Add the insecticide (i.e. from pipette or from the glass cylinder) into the mixing beaker. Pipette the mixture up and down to partly mix it but also to flush out any insecticide that might stick to the sides of the pipette tip.

4.6.8. Use the remainder of the water to rinse out the cylinder that contained the insecticide (if there is one). This is important as some insecticide may stick to the sides of the cylinder.

4.6.9. Use a plastic disposable spoon (or metal if the protocol states that this is necessary) to mix thoroughly the insecticide in the mixing beaker. Write the date, solution concentration, chemical code(s) and initials on the mixing beaker. It is likely that some solution will remain after spraying so it is important to be able to identify it.

4.6.10. If the insecticide stock is a solid (powder/granules) then use the weigh scales to measure it out before mixing with the diluent (usually deionised water). Use a metal spatula to transfer the amount of insecticide needed into a weigh boat placed on the weigh scales.

4.6.11. There may be other specific weighing and mixing instructions for particular insecticides so always check the protocol before starting the weighing.

4.7. Spraying the insecticide solutions

4.7.1. Repeat steps 4.4.1 to 4.4.11 using the insecticide solution instead of the tap water and suitable untreated blocks.

4.7.2. Start with the same volume of insecticide solution in the delivery vial as that which gave the right target block weight when you did the calibration with water. Keep the insecticide solution on the magnetic stirrer between each spray run so that it is continuously being stirred. This is important to keep weight and concentration of insecticide between the sprayed blocks uniform

4.7.3. For all the blocks within an acceptable range, write: the date of spraying, the chemical code of the insecticide that was sprayed, the test item code and the protocol number on to the block label (on the underside of the block). Record on form.

4.7.4. Move the sprayed blocks that are within range (these are now 'test items') into a temperature and humidity-controlled room.

4.7.5. For all rejected blocks, do not assign a test item code but do record them on the form. Under test item code write 'Rejected'. Use additional forms as necessary and staple them together. Rejected blocks must be put into the biohazard bags in the Spray Room for disposal.

4.7.6. Filter paper circles (cut to 8.5cm diameter with paper cutter) should be sprayed before and after each run of AI blocks i.e. two filter papers per insecticide or per concentration, or a specified in the study plan. Filter papers should be given unique numbers in the same way as the blocks but do not write these numbers directly onto the filter paper. Let the filter paper dry for one hour in the room then wrap them into aluminium foil and write the unique number onto the foil. Put the aluminium foil into sealable plastic bags and store them in the fridge until ready for dispatch to the analytical facility (GLP certified facility for GLP

studies) for HPLC/GC analysis (this is for quality control of the treatment applications).

4.7.7. If specified in the protocol, any insecticide solution that is not used for the spraying should be put into amber glass bottle(s), labelled with chemical code, spray date, solution concentration and initials. These can be sent off for chemical analysis at external institutes.

4.8. Cleaning the potter tower between insecticides, and after spraying

4.8.1. Follow steps 4.3.1 to 4.3.3 above for cleaning the Potter Tower between spraying with different AIs or different formulations and concentrations of the same AI. Also follow these steps once all spraying is done.

4.9. Cleaning and disposal of insecticide-contaminated equipment and consumables

4.9.1. Reusable apparatus and tools (beakers, cylinders, spatula etc) should be washed out thoroughly following the same procedures as for the Potter Tower.

4.9.2. Insecticide working solutions should be poured into the 20L jerry can and subsequently disposed following waste management and disposal SOP.

4.9.3. All contaminated consumables (paper towels, mixing disposable spoons, weigh boats, filter tips, coverslips etc) should be put into biohazard bags into the biohazard bin and subsequently disposed following waste management and disposal SOP.

4.9.4. Clean floor and other areas surrounding the potter tower, within the fume hood. General cleaning in the spraying section/room must be done using specific equipment, labelled and stored in that area and never used elsewhere

4.10. Potter tower maintenance

4.10.1. Clean the potter tower after spraying, including flushing the atomizer with solvent. Additionally, every month, regardless of whether the potter tower has been used or not, clean interior and exterior of the column and the tower with a paper towel dampened with water.

4.10.2. Every month, all joints and tubing should be examined for tightness and possible leaks. Use a Pasteur pipette to put a few drops of soap solution over

each joint. Run the compressor and check to see if any bubbles appear at the joints. Tighten with a wrench as needed.

4.10.3. Every month, lubricate the elevating screw (the handle at the rear of the tower) with lubrication oil for machines

4.10.4. Do not use metal objects to clear chemical deposits from the jet tips, as deforming of the orifice will result in uneven spray formations.

4.11. Collection and reporting of data

4.11.1. Ensure the following data is recorded in the data collection sheets

- Date block made
- Date of spraying
- Protocol code
- Insecticide code
- Substrate
- Number of blocks or filter papers
- Target concentration of AI
- Calculation area
- Spray weight acceptance (including test item code, target spray weight, actual spray weight, within 10% range, accepted or rejected, study director signature).

5. Health and Safety

For GLP-compliant laboratories, the following should be installed in the laboratory and field prior to Phase I IRS:

5.1. Physical structure health and safety

- Fume hood/plastic enclosure for the potter tower
- Negative air pressure in insecticide storage room and spray room
- Including ventilation fans in insecticide storage room and in spray room
- Spill kit

- Emergency shower
- Emergency eye wash

5.2. Personal protective equipment (PPE)

- Spray suit coveralls
- Respirator mask (fit-tested for the specific individual spraying)- check AI Material Safety Data Sheet for filter requirements
- Gloves
- Goggles and/or full-face Visor
- Over boots

6. Glossary of terms

A/C	Air conditioning
AI	Active Ingredient
GC	Gas chromatography
GLP	Good Laboratory Practice
I2I	Innovation to Impact
IRS	Indoor Residual Spray
KCMUCo	Kilimanjaro Christian Medical University College
PPE	Personal Protective Equipment
RH	Relative Humidity
SC	Suspension Concentrate
SOP	Standard Operating Procedure
WHO	World Health Organisation
WHOPES	World Health Organization Pesticide Evaluation Scheme

7. References

WHOPES guidelines- Testing mosquito adulticides for indoor residual spray and treatment of mosquito nets

Innovation to Impact
Pembroke Place
Liverpool L3 5QA UK

contact@innovation2impact.org
[illegible]
