# Supplementary material for: Comparative analysis of the Potter Tower and a new Track Sprayer for the application of residual sprays in the laboratory
Source: Parasit Vectors. 2024 Feb 16;17:66. doi: 10.1186/s13071-024-06168-x (PMC10870534; doi:10.1186/s13071-024-06168-x)
Supplement: Supplementary file 2 — Additional file 2. Standard Operating Procedure (SOP): Operation and maintenance of the Track Sprayer, provided by the Liverpool Insect Testing Establishment (LITE) at the Liverpool School of Tropical Medicine (LSTM). [file 13071_2024_6168_MOESM2_ESM.pdf]

SOP: Track Sprayer

July 2023

**Title**	Operation and Maintenance of the Track Sprayer
**Document number**	I2I-SOP-025
**Version number**	1
**Date first published**	
**Date last revised**	

Prepared by

Name	Role	Institution
Annabel Murphy	Research Assistant	LSTM, I2I
George Parsons	Research Assistant	LSTM
Katherine Gleave	PDRA	LSTM, I2I
Rosemary Lees	Senior Technical Officer	LSTM, I2I
Jane Bonds	Professor	Independent consultant

Timeline

Version	Date	Reviewed by	Institution
1	22/06/22	Annabel Murphy	LSTM
2			

Version Control[1]

Version	Date	Updated by	Description of update(s)

Related documents

- I2I Best Practice SOP Library, June 2023 (https://innovationtoimpact.org/)

1. Purpose

The purpose of this Standard Operating Procedure (SOP) is to provide guidelines on the set-up, calibration, operation troubleshooting guide and cleaning of the Micron Track sprayer, located on the 2nd floor of the accelerator building, room 220C.

2. Background

Located at LSTM, the Micron Track sprayer can be used to carry out initial spraying when testing the residual efficacy of insecticides on various surfaces. It is highly efficient (able to treat multiple surfaces simultaneously) and used for the application of IRS compounds onto selected surfaces including blocks made of mud, concrete, plywood and tiles for subsequent assays and enclosed within a chamber for user safety. This equipment is used

[1] Historical versions of SOPs can be found on the I2I website (https://innovationtoimpact.org/)

for the application of test compounds onto selected surfaces, enabling the study of formulations for existing and novel compounds, being developed in long lasting residual spray programs. It accurately represents IRS in the field and can assess application techniques as well as the insecticides themselves enabling Improved reproducibility and standardisation of application of insecticide to IRS-treated test surfaces (WHO., 2015). This will allow better data to be generated in the development and evaluation of vector control tools. The entire set up of the track sprayer in the Perspex chamber is shown in Figure 1.

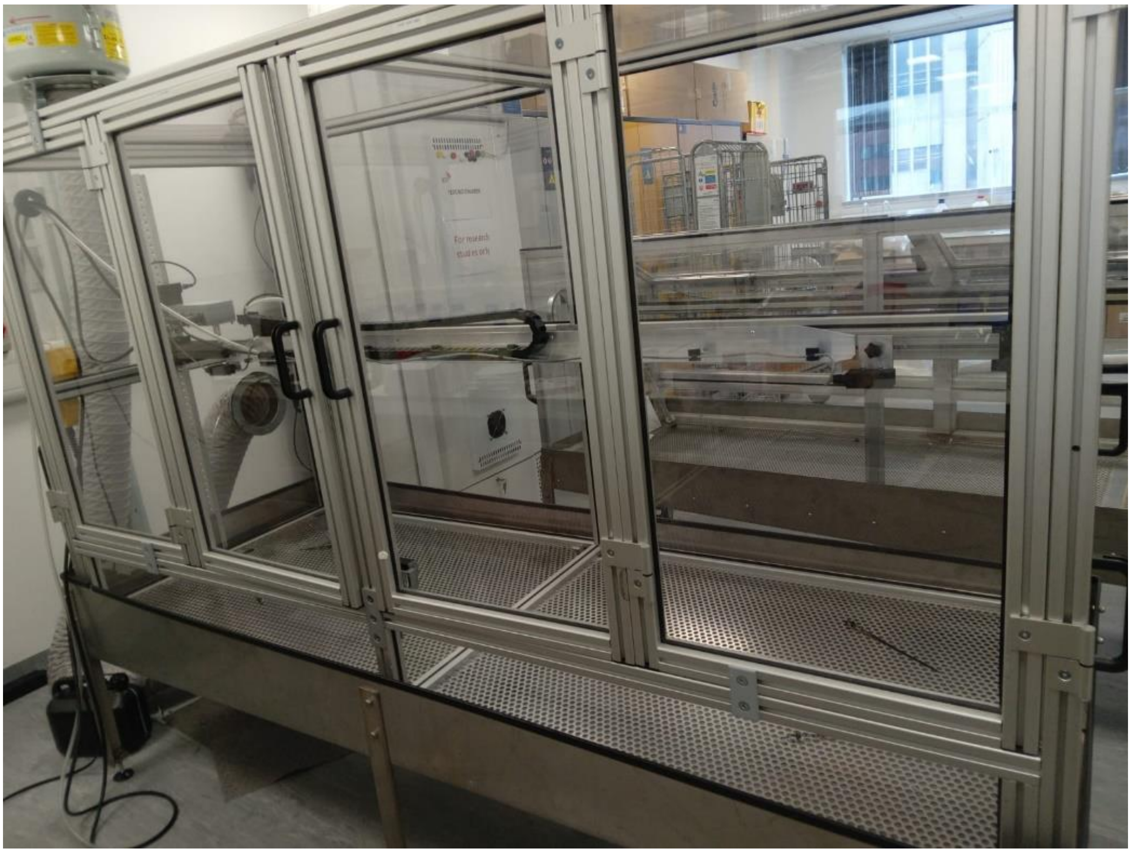

Figure 1. Set up of the track sprayer in the enclosed Perspex chamber.

3. Materials and equipment

3.1. General

- Micron Track Sprayer
- Track sprayer control panel
- Enclosed Perspex chamber
- Filtermist (air filter)
- Test surfaces
- Battery
- Blue roll

3.2. Required for calibration

- Fluorometer (Trilogy™ or equivalent)
- Fluorescein sodium salt
- Pipette
- Cuvettes
- Distilled water
- Blue roll
- Petri dishes
- Cover slips
- Zip lock bags

3.3. Required for cleaning

- Decon 90 (5%)
- Cleaning bucket
- 500 mL bottle
- Measuring cylinder dedicated to Decon 90
- Tap water
- Blue roll
- Yellow clinical bio-hazard waste bags and cable ties
- Hose and sprinkler nozzle (attached to tap)

4. Procedure

4.1. Installation of Micron Track Sprayer

- Ensure the enclosed Perspex chamber is intact and clear of other equipment and lift into the chamber.
- Requiring two people at either end carry, lift and insert the track sprayer into the enclosed Perspex chamber.
- To secure the track sprayer into place lift it to where "number 10" is written on the vertical metal poles, as indicated in Figure 2. This creates a fixed distance of 45cms between the spray nozzle and the top of a petri dish sitting on the floor, which will be the distance during calibration. The track can be raised in increments of 2cm for any surfaces which are raised higher, enabling a distance of 45cm to be consistently maintained throughout spraying.
- Once in position, lock it in place using the metal pin attached to the vertical metal pole. Do this for both sides of the track sprayer as shown in Figure 3 and 4.
- Set up the ventilation system by attaching the filter tubing to the Perspex enclosure (Figure 5).
- Plug in the battery into the nozzle control unit using the black lead as shown in Figure 6.
- Attach the inlet pipe directly to the top of the stainless compression sprayer as shown in Figure 7.

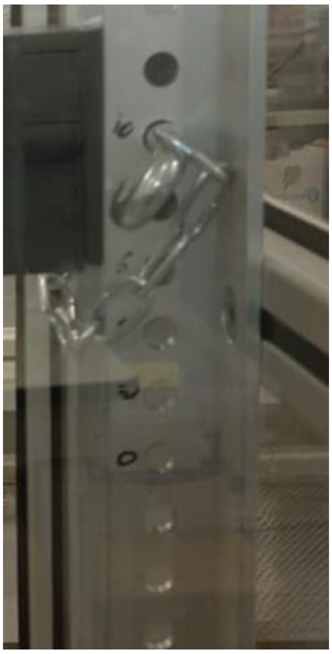

Figure 2: metal vertical poles with different numbered positions

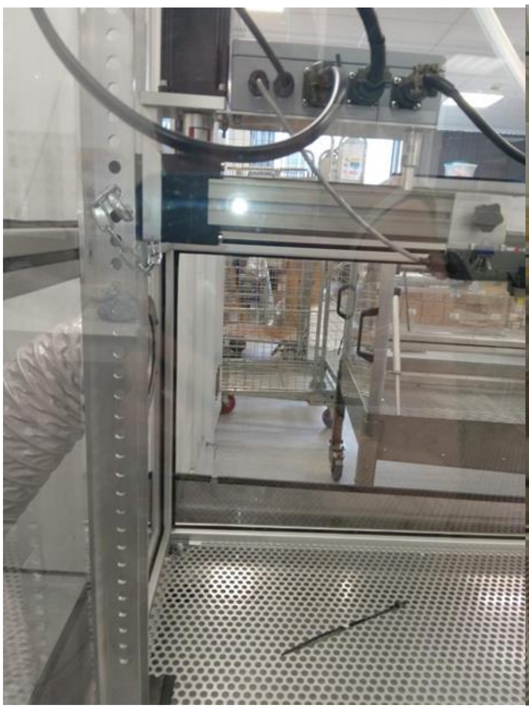

Figure 3 Left hand side of the track sprayer

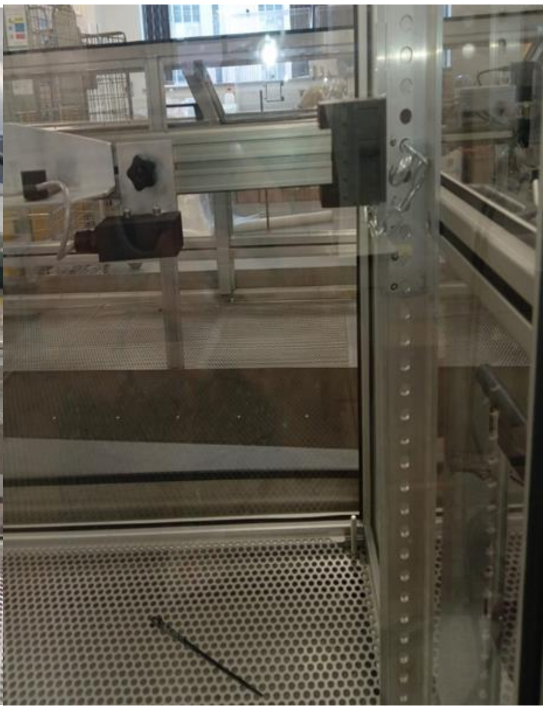

Figure 4: Right hand side of the track sprayer

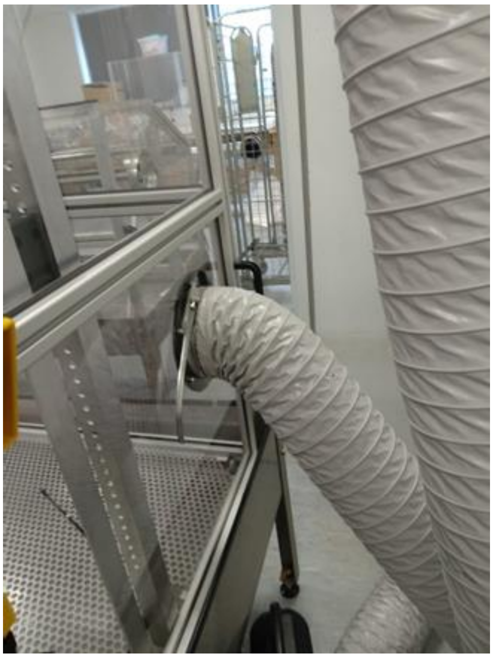

Figure 5: Tubing attached to filtermist and enclosure

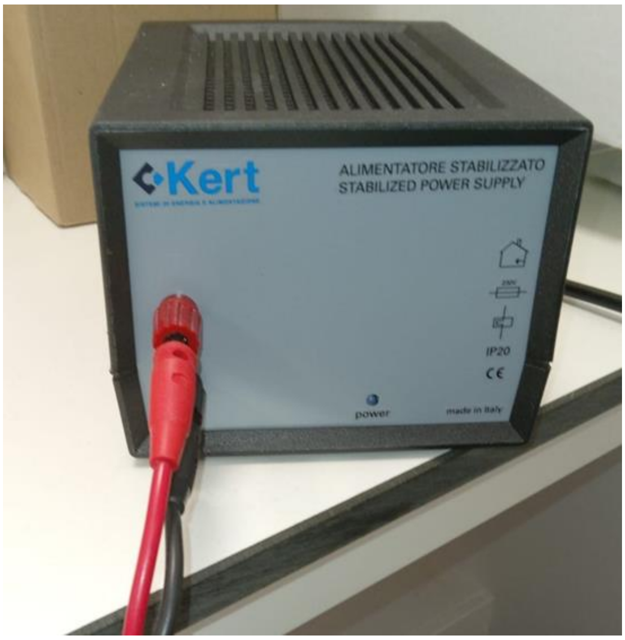

Figure 6: Battery attached to nozzle control unit.

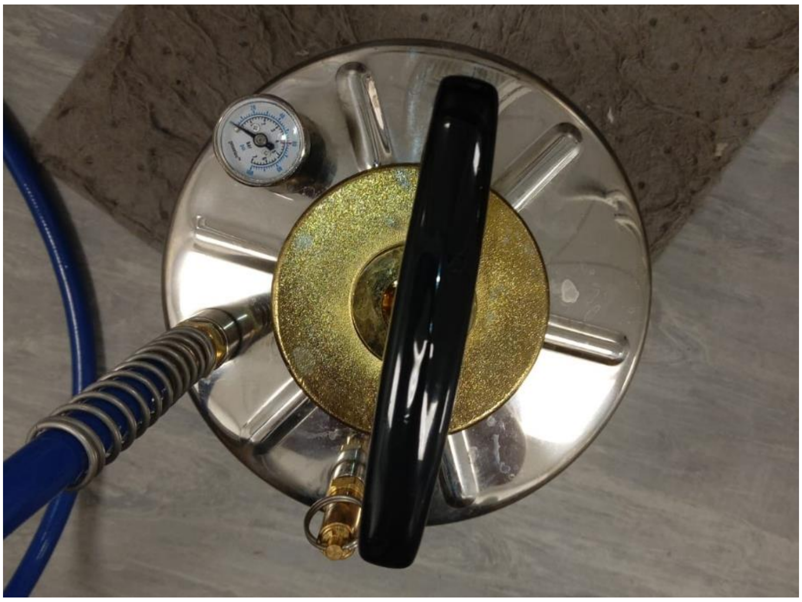

Figure 7: Inlet pipe inserted directly to the top of compression sprayer.

4.2. Procedure for making up test compounds

This section details the procedure required for making up the spray product in the laboratory. Formulation insecticides which are water dispersible are suitable for use in the track sprayer. The minimum volume required for the Track Sprayer is 500mL. Solvents (e.g., acetone which are required to dissolve active ingredients) are not used due to health and safety concerns. To measure out solid and liquid insecticides use the following steps.

- To weigh out a solid insecticide, in the fume hood, obtain the glass bottle for the stock, label and tare it on the balance. Using a spatula, carefully weigh out the amount of insecticide required.
- Measure out the correct quantity of distilled water in a funnel. Dilute the formulation by slowly pouring this into the bottle containing the weighed-out insecticide.
- Add and secure the bottle cap, gently agitate until satisfied the granules have fully dissolved.
- To measure out a liquid insecticide, In the fume hood carefully pour the insecticide into a funnel up to the desired quantity and pour this into the glass bottle. The liquid insecticide should be measured in grams not mililitres and converted volumetrically using supplied density information.
- Measure out the correct quantity of distilled water taking into account the density of the liquid insecticide.
- Add to the bottle containing the insecticide and wash down any insecticide still in the funnel using some of this distilled water and add it to the bottle.
- In a separate funnel. Add to the glass bottle containing the insecticide, wash down any insecticide still in the funnel using some of this distilled water and add it to the bottle.
- Add and secure the bottle cap, gently agitate until satisfied its dissolved.
- To calculate the quantity needed for the desired application rate refer to Table 1.

Table 1: Calculation of Insecticide Quantity per Tank Load. Actellic formulation (300CS) applied at 1g/m² using a 500mL tank load

**A**	Dose of the insecticide active ingredient to be applied in gram per square meter.<br><br>(Refer to product for quantity of active ingredient per square meter. If the value is given in mg convert to grams by dividing by 1000.)	$1g/m^2$
**B**	Percentage of the insecticide formulation being used<br><br>(Refer to packet label for this information e.g., 300/L=30%)	30%
**C**	Amount of water in the sprayer tank in milliliter (mL)<br><br>(A minimum of 500mL is required in the compression tank)	500mL
**D**	Amount of liquid suspension applied per m^2<br><br>(Flat fan CFV applies a rate of $30mL/m^2$)	$30mL/m^2$
**E**	Surface to be treated with one tank load = C ÷ D =<br><br>(Divide the value for C by D to get a value in square meters)	$500/30=16.67m^2$
**F**	Quantity of active ingredient needed to cover wall area = A x E grams<br><br>(Multiply the value for A by the value for E to get a value in grams)	1x16.67=16.667g
**G**	Quantity of formulation needed per tank load to cover area<br><br>= F x (100/B) mL<br><br>(Divide 100 by the value for B and multiply it by the value in F to get a value.)	16.667 x (100/30)<br>=<br>**55.5mL**

4.3. Calibrated settings

These settings must be inputted for every spray application. The nozzle is calibrated to achieve a desired application rate of $30ml/m^2$, using the settings: Speed:0.45 (m/sec). The

calibrated settings set for speed are determined using the fluorometry method detailed in 'Procedure for calibration of spray output' and the speeds is set following the application rates obtained in this method.

4.4. Procedure for application of test compounds onto surfaces

- Prior to spray application ensure the following:
- Both the chamber doors should be fully closed.
- Wiring should not be obstructing the nozzle during spraying: tape into place if required.
- Ensure that all air bubbles are clear within the tubing by purging the solution.
- Check all tubing and wire connections for visual damage and ensure there are no leaks.
- Visually inspect the nozzle, if a tilt is identified, carefully adjust the nozzle manually by hand until nozzle is centralised.
- Check the nozzle to ensure there is no clogging (if clogging is suspected refer to the troubleshooting guide) If necessary, replace the nozzle.

4.5. Operation for spray application

- Place the surfaces to be treated on the track sprayer floor. Surfaces should have petri dishes underneath to treat the test substrate to raise it the 1.2cm so that the height between calibration and test spray are consistent.
- Remove pressure in the compression tank by pulling the release valve. Open the lid and insert the spray solution in the tank, a minimum of 500mL and maximum of 2 Litres.
- Operate the pump using both hands until the tank pressure reaches at least 40 psi on the pressure gauge.

- Nozzle speed is controlled by the control unit where a traverse speed between 0.2-0.75 (m/sec). Set the speed to 0.45m/s (or the correct speed achieved in calibration) (Figure 8). Ensure the control panel is switched off by ensuring the red safety button is fully pressed down. Set the speed of the track sprayer in m/s by turning the knob on (Figure 8), anti-clockwise to decrease and clockwise to increase.
- Select the function 'both' on the control panel. This enables movement of the nozzle whilst simultaneously spraying.
- Select the function 'both' on the control panel. This enables movement of the nozzle whilst simultaneously spraying.
- Using the switch labelled 'direction' control the direction of the sprayer.
- To begin spraying and movement of the nozzle. Release the red safety stop button on the track sprayer control unit. Twist clockwise until you hear a click (Figure 11).
- To prevent draining the battery press down on the red safety button to completely switch off.
- Turn the extractor fan on by pressing the green button in Figure 9. Allow to run for 30 seconds.
- Return the sprayer back towards the control panel by selecting traverse only. Always carry out this step after the surfaces have been removed to prevent any accidental deposition of spray solution from the nozzle.
- Repeat until all the required surfaces have been treated with that spray solution. Check that the pressure is still minimum 40 psi on the pressure gauge for the next spray, if not increase the pressure.
- If a higher concentration of the same product is going to be sprayed, cleaning is not required.
- If a new compound is going to be sprayed next, the Micron Track Sprayer and associated apparatus and enclosure must be cleaned with decon90 and water.
- Cleaning must be performed at the end of each stock solution as detailed in the cleaning and maintenance section.

- To prevent draining the battery press down on the red safety button to completely.

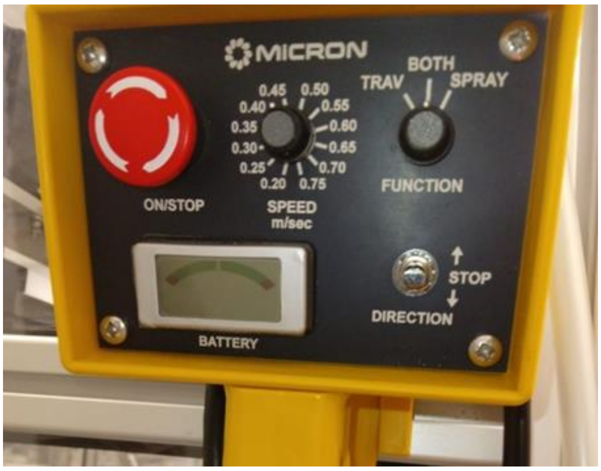

Figure 8: Control Unit

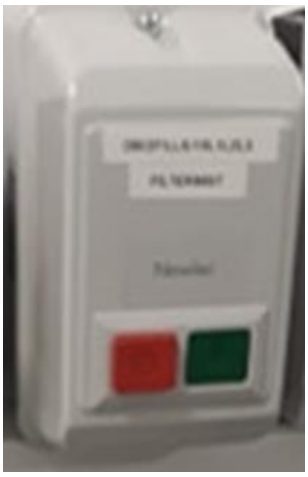

Figure 9: Filtermist control

4.6. Procedure for Calibration of Spray Output

The following procedure calibrates the rates of spray deposition (ml/m^2) required to deposit the desired application rate onto the treated surface. This should take place routinely and prior to a project. When making up spray solutions, approximately 500ml should be made up for this procedure. The following steps detail preparation of the dye concentration standards and application of the spray deposits using the track sprayer.

- Generate a calibration curve by making up the reagents shown in Table 3.
- Add the stock solution to the compression pump and spray directly from the nozzle into a 50mL falcon tube to obtain at least 10mL of stock solution.

- To prepare a set of standards from the stock solution, take 5, 10, 20 and 30 uL of the stock solution and dilute in 10 ml of the extraction solution, as detailed in Table 4.
- Follow the steps outlines in Table 4.
- Add 1-2mL of the solution from each standard into a cuvette and place into the fluorometer. (For use with Trilogy fluorometer) Select "blue module" and "measure fluorescence raw" to obtain raw fluorescence units (RFU).
- Using these values, in Excel (spreadsheet to be made available from LSTM) create a calibration curve of RFU (y axis) against µl concentration (x axis), determine the gradient by adding the equation of the curve. From each subsequent reading, subtract the blank RFU value and divide by the gradient. This value can be later transformed to give application rate in ml/m^2.
- A new Calibration Curve should be generated for each new extraction solution and sample stock solution directly from the nozzle directly before or after spraying on surfaces. A new calibration curve could be required per run.
- Open both chamber doors of the enclosure.
- Obtain 8 petri dishes, discard the lid and place upside down on the floor of the sprayer to have 4 in both sections of the track sprayer, as shown in Figure 10.
- Using the stock solution of fluorescein made up operate the track sprayer to spray the petri dishes.
- Collect dishes carefully by holding the sides between thumb and finger and place them into plastic sealable bags of at least 13 x 13 cm.
- Measure 100 mL extraction solution in a graduated cylinder or using a larger pipette and add to each bag.
- Agitate the bags so that the whole of the inside of the bags has been covered by extraction solution.
- Pour out approximately 1-2 mL of the solution into cuvettes (by eye is fine, volume depends on size of cuvette) and operate the flurometer to obtain RFU values.

- Petri dishes and bags may be reused if rinsed 3 times with water and dried. If cuvettes are being re-used, rinse out three times with distilled water and again a further three times with distilled water and a bottle brush.
- If the volume of sprayed product (ml/m2) is not accurate to the required volume within an acceptable range of 10%, follow the "checking validation parameters" section in this SOP.
- Adjust accordingly and repeat until the correct rate of deposition has been obtained. Make a recording of the final application rate achieved.

Table 3: Reagents required for generating a calibration curve

Solution	Procedure
Prepare 1 litre of extraction solution	Add 100mL of NaOH to 900mL of dionsied water. Add 1mL of Tween 20
Prepare 1 Litre stock solution of 0.005% fluorescein sodium salt	Dissolve 0.05g of fluorescein sodium salt into 1L of deionised water. Add in 1mL of Tween 20

Table 4: Standard preparation

Total Volume	Stock volume (µl)	Diluent extraction solution (distilled water + 0.1% Tween20)
10 mL	0 (Blank)	10 mL
10 mL + stock	5	10 mL
10 mL + stock	10	10 mL
10 mL + stock	20	10 mL
10 mL + stock	30	10 mL

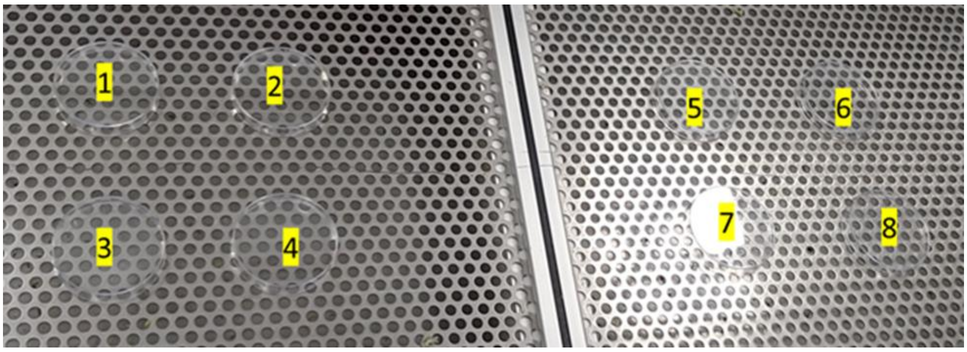

Figure 10: Layout of petri dishes within the Horizontal Track Sprayer

4.7. Checking validation parameters

This section details checks that should be carried out at the start of each spraying session, i.e. before any surfaces are sprayed. These validation parameters are detailed below.

Parameter	Method
Centralisation of the spray nozzle	• Visually inspect the nozzle<br>• If a tilt is identified, carefully adjust the nozzle manually by hand until nozzle is centralized.

Spray distribution	• Place blue roll across and along the swatch of the track sprayer at (insert exact measurements).<br>• Apply spray solution/water at the calibrated settings, following steps detailed in the 'procedure of application of test compounds onto a surface.'<br>• Visually inspect the blue roll to ensure the spraying pattern is even and there are no visible deviations of distributed insecticide.
Flow rate	• Use water as the spray solution. Ensure the control panel is off, set the speed, select 'spray'.<br>• Hold a graduated beaker in place under the spray output of the nozzle.<br>• Release the safety button of the control unit to begin spraying. Start a stopwatch straight away.<br>• After one minute on the stopwatch stop spraying.<br>• Measure the amount of liquid collected to obtain the ml/min value.

4.8. Troubleshooting

If results from fluorometry are inconsistently out/highly variable between or within reps check the following.

Parameter	Method
Check the speed of the carriage against the control panel (m/s)	• Mark out a metre on the track. This can be done by applying masking tape on the track, measuring and scoring 1 metre with a black marker.<br>• Ensure the control panel is switched off by pressing the red safety button down. Set the speed of the track sprayer in m/s<br>• Select the function ‘trav’ on the control panel and select ‘up’ to move the spray at the selected speed away from the control panel. Note: it is more accurate to do this at a low speed.<br>• Set up a stopwatch to record time in seconds. To begin movement of the nozzle. Release the red safety stop button on the track sprayer control unit.<br>• Begin the stopwatch as soon as the carriage passes 0 on the marking and stop as soon as it reaches 1m.<br>• Calculate the speed in m/s using the calculation: **Speed=distance/time (seconds)**<br>• Repeat this process three times and take an average.<br>•
Nozzle spray	Deviation of sprayed distribution onto blue roll suggest there’s a potential clog in the nozzle. Clean by:<br>• Put the nozzle in a container and immerse in water. Leave for several hours.<br>• Remove the blockage with a small scrubber (see Figure 11.)<br>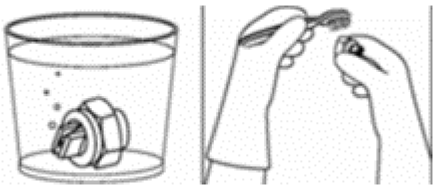<br>*Figure 11: nozzle being cleaned*

Pulsing of spray	• Flush some solution through the system and check it is consistently spraying and not pulsing (this is where the spray solution will stop and start during output). If pulsing is occurring more solution may be required in the compression tank. Ensure that all air bubbles are clear within the tubing by squeezing the air bubble and/or purging the solution.

4.9. Cleaning and Maintenance

The following section describes the cleaning process for the Micron Track Sprayer and enclosed Spray Cabinet. Cleaning after the application of insecticide treatments is essential to keep the equipment in good condition and to ensure all residual insecticides are removed at the end of each test performed, preventing contamination to future treatments. The scope of this procedure covers the following, the tubing within the Micron Track Sprayer, stainless compression tank spray cabinet enclosure, spray table racking, nozzles. This procedure identifies the correct sequence of activities and provides a standard, consistent cleaning method to perform as well as the reagents and concentrations required. The cleaning schedule is conducted a Post use of the track sprayer. This is to ensure all equipment is well maintained, decontaminated and ready for future use. And between the use of different insecticide formulations.

- To clean the stainless steel compression tank and tubing follow these steps.
- Pre-clean: Turn on the filtermist extractor fan and purge the tubes of the sprayer of any insecticide from previous testing, until satisfied they are clear.
- Depressurise the tank using the decompression switch, remove the lid.
- Discard by pouring any remnants of insecticide in the tank into the track sprayer channel at the bottom of the chamber.

- Clean: Rinse 1 (stainless compression tank**):** Add 500mL of tap water into the tank using a portable container, swirl and discard into the drainage channel at the bottom of the chamber, into the grid. Repeat three times.
- Rinse (tubing): Using tap water fill the tank to 1 Litre. Pressurise and allow the sprayer to run, purging off all the water through the tubing.
- Add 500mL of 5% Decon 90 (refer to LITSOP046 Cleaning of Laboratory Areas in LITE) to the tank, pressurise, and purge the tubes of the sprayer containing the Decon 90, Visually confirm that the tubes are clean.
- Depressurise the tank and remove the lid. Discard any remaining Decon 90 into the track sprayer drainage channel, at the bottom of the chamber, into the grid. Repeat rinse 1.
- Post clean: Turn off the filter mist extractor fan. Turn the control panel of the track sprayer off.
- Next clean the spray cabinet and spray table racking. Be careful not to apply any solution to the electrical components. A different rinsing method is used for the spray table racking and the Perspex walls of the spray cabinet enclosure to prevent this.
- Clean: Rinse (spray table racking): Attach the hose and sprinkler nozzle to a cold water tap. Rinse down the spray table racking only. Visually inspect to ensure there are no visible traces of insecticide.
- Rinse (Perspex walls): Fill a 500ml container with tap water. Rinse down each interior wall ensuring all areas are covered.
- Spray all areas of the interior walls and spray table racking with the Decon 90 (refer to LITSOP046 Cleaning of laboratory Areas in LITE). Check all hard to reach internal surfaces are in contact with the cleaning solution.,
- Wipe the surfaces of the interior walls with blue roll using overlapping strokes to ensure that no parts of the surface are missed. Used blue roll must be placed in the appropriate waste bin. (Refer to LITSOP008 Waste Disposal).
- Leave to soak for a total contact time of 30 minutes with the Decon 90 solution.
- On completion of the minimum soak time carry out the following:
- For the spray table racking and Persepx walls: Repeat rinse three times.

- To clean the nozzle wipe down with blue roll soaked in 5% Decon 90 followed by blue roll soaked in water. Allow to air dry and visually check for no remnants of insecticide.

4.10. Health, Safety and Environmental Protection

Ensure that prior to using any chemical or biological compound all associated Risk Assessments (RA) and Material Safety Data Sheets (MSDS) have been read and understood, training must be documented in the individuals' training file.

Refer to Control of Substances Hazardous to health (COSHH) Assessment for each chemical used.

All staff working in LSTM laboratories must have received LSTM induction training.

All staff using this procedure must be trained in safe operation of chemical fume hoods, refer to LITSOP074.

Dispose of all waste materials appropriately as laid out in LITSOP008 Waste Disposal.

The appropriate PPE, determined by the RA for the product being sprayed, should be worn throughout handling the insecticides and application of treatment to the surfaces. Personal Protective Equipment (PPE) as specified in the RA must be available, and worn during preparation, using or disposing of the Decon90 cleaning solution.

Avoid entering the track sprayer room without full respiratory protection for one hour post spray. This is to allow time for air borne contaminants to be removed. It is advised to wear a 3M versflow powered hood respirator (M306) with a particulate and nuisance organic vapour filter (TR 3802E) when working with aerolised chemicals.

Check any electrical equipment to be used has no visible signs of damage to the wiring, plug, or any components prior to use. If damage is observed do not use and report to

manager/supervisor. Do not leave the battery charging for more than 8 hours. Check the expiration date of the filtermist filter.

5. Additional data collection

6. Deviations from standard protocol

The following procedure details an alternative method that can also be used to calibrate the track sprayer, using cover slips instead of petri dishes. It details the preparation of the dye concentration standards and application of the spray deposits using the track sprayer.

- Generate a calibration curve by making up the reagents shown in Table 3.
- Add the stock solution to the compression pump and spray directly from the nozzle into a 50mL falcon tube to obtain at least 10mL of stock solution.
- To prepare a set of standards from the stock solution, take 5, 10, 20 and 30 uL of the stock solution and dilute in 10 ml of the extraction solution, as detailed in Table 4.
- Follow the steps outlines in Table 4.
- Add 1-2mL of the solution from each standard into a cuvette and place into the fluorometer. (For use with Trilogy fluorometer) Select "blue module" and "measure fluorescence raw" to obtain raw fluorescence units (RFU).
- Using these values, in Excel (spreadsheet to be made available from LSTM) create a calibration curve of RFU (y axis) against µl concentration (x axis), determine the gradient by adding the equation of the curve. From each subsequent reading, subtract the blank RFU value and divide by the gradient. This value can be later transformed to give application rate in ml/m^2.
- A new Calibration Curve should be generated for each new extraction solution and sample stock solution directly from the nozzle directly before or after spraying on surfaces. A new calibration curve could be required per run.
- Open both chamber doors of the enclosure.

- Place 9 upside down petri dishes on top of the spray table. The exact positions are marked on the metal grid of the spray table. The layout consists of three rows across and along the swath, as shown in Figure 12.
- Place a small piece of blue roll on top of each petri dish.
- Place cover slips on top of each petri dish. One is required for testing the flat fan nozzle.
- Using the stock solution of fluorescein made up operate the track sprayer at the calibrated settings.
- Using forceps grip the corner of each cover slip and place into a 25mL plastic pot (a separate pot is used for the cover slips on each petri dish).
- Pipette 10mL of water into each plastic pot, close and agitate to ensure all fluorescein is dissolved.
- Operate the fluorometer. Continue until an RFU value is obtained for all the cover slips and repeat three times to obtain an average. If cuvettes are being re-used, rinse out three times with distilled water and again a further three times with distilled water and a bottle brush.
- Adjust accordingly and repeat until the correct rate of deposition has been obtained. Make a recording of the final application rate achieved.

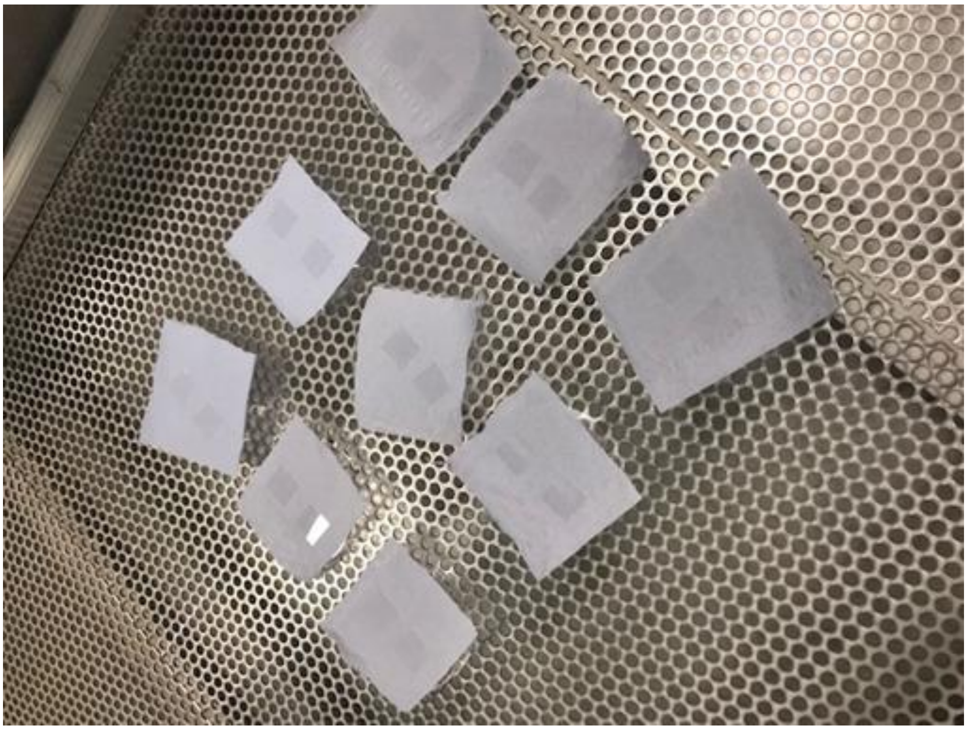

Figure 12: Cover slip set up in the track sprayer

7. Glossary of terms

**SOP**	Standard Operating Procedure
**LITE**	Liverpool Insect Testing Establishment
**RA**	Risk Assessment
**LSTM**	Liverpool School of Tropical Medicine
**CFV**	Control Flow Valve
**MSDS**	Material Safety Data Sheet
**PPE**	Personal Protection Equipment
**IRS**	Indoor Residual Spraying

**TFM**	Test Facility Manager
**m/s**	Metre per second
**COSHH**	Control of substances hazardous to health
**m^2**	Metre squared
**mL**	mililitres
**min**	Minute
**Psi**	Pound-force per square inch
**Mm**	Milimetre
**w/v**	Weight in volume
**G**	gram
**L**	Litre
**m**	Metre
**RFU**	Relative fluorescence units
**mL/min**	Millilitres per minute

8. References

Referenced Documents

ITFSOP008	Waste Disposal
LITSOP027	Deviation management
LITSOP074	Operation and Maintenance of the Mach Aire Fume Cupboards
ITFRA006	Decon 90 Solution
ITFRA008	Ethanol Solution
ITFRA056	Cleaning and Decontaminating
LITSOP046	Cleaning of laboratories and Associated Areas in LITE
ITF/RA/062	Waste Disposal of Insecticides
ITF/RA/006	Decon 90 Solution
ITF/RA/056	Cleaning and Decontaminating
LITERA351	Mach Aire Fume Cupboards

Bonds, J., Parsons, G., Walker, K. J., Lees, R. S., Murphy, A., Malone, D., & Foundation, M. G. (2023). Comparative analysis of the Potter Tower and a new Track Sprayer for the application of residual sprays in the laboratory. *Preprint*, 1–21. Retrieved from https://assets.researchsquare.com/files/rs-2598764/v1/e3c3bf06-3c59-4b67-b93e-1a7a09fc44d0.pdf?c=1677080217

World Health Organization., (2015). *An Operational Manual for Indoor Residual Spraying (IRS) for Malaria Transmission Control and Elimination*.
