# Supplementary material for: Correction: Comparative analysis of the Potter Tower and a new Track Sprayer for the application of residual sprays in the laboratory
Source: Parasit Vectors. 2026 Mar 7;19:104. doi: 10.1186/s13071-026-07272-w (PMC12967721; doi:10.1186/s13071-026-07272-w)
Supplement: Supplementary file 1 — Additional file 1. [file 13071_2026_7272_MOESM1_ESM.pdf]

Bonds *et al. Parasites & Vectors (2024) 17:66*
https://doi.org/10.1186/s13071-024-06168-x

Parasites & Vectors

RESEARCH

Open Access

Comparative analysis of the Potter Tower and a new Track Sprayer for the application of residual sprays in the laboratory

Jane Bonds[1], George Parsons[2], Kyle J. Walker[2], Annabel Murphy[2], Rosemary Susan Lees[2,3*], Derric Nimmo[4], John Clayton[5] and David Malone[6]

Abstract

Background Efforts to evaluate the residual efficacy of new indoor residual spraying (IRS) formulations have identified limitations with the industry standard laboratory sprayer, the Potter Spray Tower (PT). Calibrating the PT can be time-consuming, and the dosing of surfaces may not be as accurate or uniform as previously assumed.

Methods To address these limitations, the Micron Horizontal Track Sprayer with Spray Cabinet (TS) was developed to provide higher efficiency, ease of operation and deposition uniformity equal to or better than the PT. A series of studies were performed using a fluorescent tracer and three IRS formulations (Actellic® 300CS, K-Othrine WG250 and Suspend PolyZone) sprayed onto surfaces using either the PT or the TS.

Results Deposition volumes could be accurately calibrated for both spray systems. However, the uniformity of spray deposits was higher for the TS compared to the PT. Less than 12% of the volume sprayed using the PT reaches the target surface, with the remaining 88% unaccounted for, presumably vented out of the fume hood or coating the internal surfaces of the tower. In contrast, the TS deposits most of the spray on the floor of the spray chamber, with the rest contained therein. The total sprayed surface area in one run of the TS is 1.2 m^2, and the operational zone for spray target placement is 0.7 m^2, meaning that 58% of the applied volume deposits onto the targets. The TS can treat multiple surfaces (18 standard 15 × 15 cm tiles) in a single application, whereas the PT treats one surface at a time and a maximum area of around 0.0225 m^2. An assessment of the time taken to perform spraying, including the setup, calibration and cleaning, showed that the cost of application using the TS was around 25–35 × less per tile sprayed. Standard operating procedures (SOPs) for calibration and use of both the Potter Tower and Track Sprayer have been developed.

Conclusions Overall, the TS represents a significant improvement over the PT in terms of the efficiency and accuracy of IRS formulation applications onto test substrates and offers a useful additional tool for researchers and manufacturers wanting to screen new active ingredients or evaluate the efficacy of IRS or other sprayable formulations for insect control.

Keywords Insecticide residual spray (IRS), Application technology, Vector control, Potter Tower (PT), Micron Track Sprayer (TS)

*Correspondence:
Rosemary Susan Lees
rosemary.lees@innovation2impact.org
Full list of author information is available at the end of the article

© The Author(s) 2024. **Open Access** This article is licensed under a Creative Commons Attribution 4.0 International License, which permits use, sharing, adaptation, distribution and reproduction in any medium or format, as long as you give appropriate credit to the original author(s) and the source, provide a link to the Creative Commons licence, and indicate if changes were made. The images or other third party material in this article are included in the article's Creative Commons licence, unless indicated otherwise in a credit line to the material. If material is not included in the article's Creative Commons licence and your intended use is not permitted by statutory regulation or exceeds the permitted use, you will need to obtain permission directly from the copyright holder. To view a copy of this licence, visit http://creativecommons.org/licenses/by/4.0/. The Creative Commons Public Domain Dedication waiver (http://creativecommons.org/publicdomain/zero/1.0/) applies to the data made available in this article, unless otherwise stated in a credit line to the data.

Background

Indoor residual spraying (IRS) is one of the primary vector control interventions for interrupting and reducing malaria transmission. IRS is the application of long-lasting, residual insecticide formulations to potential malaria vector resting surfaces such as the internal walls, eaves and ceilings of structures where such vectors might alight [1]. IRS and insecticide-treated bed nets (ITNs) account for almost 60% of global investment in malaria control [2]. In recent years, however, there has been an increase in resistance of malaria vectors to insecticides, particularly to pyrethroids, which, until recently, have been the only insecticides used in ITNs. The World Health Organization (WHO) list of pre-qualified products for vector control includes IRS products spanning five insecticide classes [3]. Nevertheless, as resistance increases, the development and evaluation of new insecticides and application strategies are urgently needed to sustain efforts to drive malaria transmission to zero [4]. The Innovative Vector Control Consortium (IVCC) works with the Liverpool Insect Testing Establishment (LITE) in the Liverpool School of Tropical Medicine (LSTM) to screen compounds for activity against mosquitoes [5] and to test formulations of promising active ingredients (AI) [5–7] to work towards IVCC's target of launching three new public health insecticides [8].

The initial testing of new IRS formulations is typically conducted within a laboratory environment. It requires controlled and precise dosing of a representative substrate (e.g., tiles, cement and mud) with the IRS formulation to which target organisms are exposed. The current WHO standard for laboratory applications of IRS formulations is the Potter Spray Tower® [9] (Fig. 1a). The Potter Spray Tower (PT) was developed at Rothamsted Experimental Station, Harpenden, Herts, England [10], and is internationally recognised by the public health community as the most precise method of chemical spraying in the laboratory (Burkard Manufacturing Co., Ltd., Hertfordshire UK). Its use is intended to provide a homogeneous deposit of the desired concentration of AI per unit area. There are, however, limitations to the PT: it can take a long time to calibrate, only one surface can be treated at a time, and the accuracy of the dosing and the uniformity of the deposit are now in question. The standard operating procedure (SOP) implemented at LITE (available online at https://innovationtoimpact.org/resources/ and as Additional file 1) includes two calibration steps prior to using the PT. The first step is to adjust the position of the spray nozzle until the weight of spray droplets deposited onto four coverslips placed centrally on the spray table deviates by ≤ 10% from the mean spray weight. The second calibration step is to check that the required spray weight is deposited over the sprayed area within a margin of ± 5% of the target weight. Once these calibration criteria have been met, the application of IRS formulations to the test surfaces can proceed. No additional calibration checks are carried out during treatment applications since calibration can take a considerable time, and it would be impractical to repeat it. Consequently, there is no confirmation that the PT maintains this uniformity throughout the treatment period.

With the continuing need to evaluate IRS formulations containing novel insecticides and evidence that there were significant limitations with the PT, we commissioned the development of a more reliable, efficient alternative. Whereas pyrethroids give rapid knockdown and kill at relatively low application rates, new compounds in development may have an endpoint, such as delayed mortality or sterilisation, which may be more sensitive to deviations from target application rates. Therefore, it is

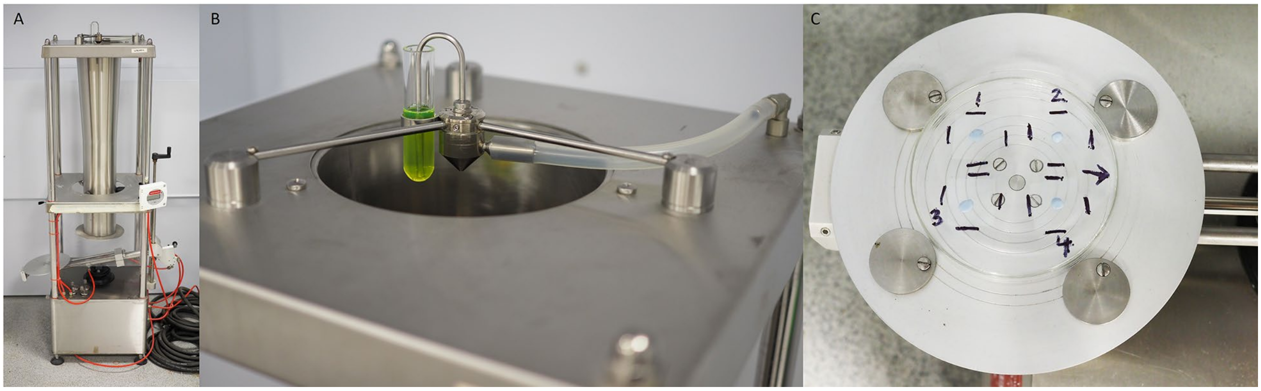

Fig. 1 The Potter Tower. The Potter Tower is shown (**A**) with a close up on the nozzle and three arms with the adjustment screws on the three stands (**B**), and the coverslip positioning on the spray table (numbered 1–4), raised on Bostik Blu Tack®, with four moveable discs to secure the Petri dish (**C**)

more important than ever that test surfaces are treated accurately to minimise the effect of variability in application rates on bioassay data. An ideal replacement would be a dedicated device with throughput, ease of use, and accuracy and uniformity of the applied dose equal to or better than the PT. The Micron Horizontal Track Sprayer with Spray Cabinet has been developed for this purpose (Micron Sprayers Ltd., Herefordshire UK). The track sprayer (TS) utilises the same conventional Flat Fan nozzle (FF 80 02E), Controlled Flow Valve (CFV) and pressurised spray tank as the backpack sprayers used for IRS application during operational deployment, so it has the additional advantage of replicating the way IRS products are applied during spray campaigns. The nozzle is mounted on a motorised track along which it moves at set speeds to deliver the required spray volume to surfaces placed on the floor of the cabinet. The height of the nozzle above the target surface can also be adjusted. The nozzle is connected to a pressurised spray tank by a length of tubing. With constant speed and uniform nozzle pressure (by use of a CFV) during application, results from the track sprayer should be highly reproducible and allow the treatment of multiple surfaces simultaneously. In addition, the TS has the capacity to produce treated surfaces to test not only chemicals but also the application techniques used to apply them. Snetselaar, Lees et al. [11] evaluated a similar system mounted vertically, which produced more repeatable deposits on surfaces in experimental huts compared to a manual backpack sprayer.

This study aimed to compare the PT and TS in three ways. First, observations of the operation and ease of use of the two pieces of equipment were made to facilitate comparison by recording the time taken for calibration, treatment and cleaning procedures. Second, the accuracy of the two pieces of equipment was determined by measuring the volume of spray treatment deposited relative to the target application rate, uniformity of that deposit and residual efficacy of the treated test surfaces. Lastly, the dose of AI deposited by both devices was assessed using high-performance liquid chromatography (HPLC).

Methods

Potter Tower calibration

The pressurised misting nozzle of the PT must be adjusted to produce a uniform spray onto the substrate positioned on the centre of the spray table. Liquids pipetted into the delivery vial at the top (Fig. 1b) are drawn through an atomiser by compressed air (15–20 psi). The gas pressure is checked and adjusted if necessary, and the guide screws are positioned to secure the Petri dish. The first calibration step is to level the tower and the nozzle arms via the adjustable feet, using the integrated spirit level, alongside a visual assessment that the nozzle is centralised. Once the device is level, the nozzle must be centralised within the tower to apply the chemical uniformly across the spray table. Four targets (deposition samplers) are placed on the spray table, and the amount deposited on each is measured by weight using a mixture of water and glycerol (50:50) to minimise losses due to evaporation compared to water alone.

The deposition samplers (cover slips) rest on pieces of Bostik Blu Tack® to hold them away from the surface (Petri dish) to prevent additional wicking of liquid from the sprayed surface (Fig. 1c). The amount of spray deposited onto each deposition sampler should be similar if the nozzle is correctly centralised. Uneven distribution can be resolved by adjusting the height of the arms that hold the nozzle. Where deposition is highest, the nozzle arm is also high on that side, so the nozzle is tilted away by lowering that arm or raising the two opposite arms. This manipulation of the three nozzle arms may require several attempts to achieve uniformity as it is not intuitive to adjust accurately; a four-arm system would have been more straightforward.

The initial weight of each deposition sampler is recorded, and they are positioned to form a square in the centre, with 1–4 always in the same position (Fig. 1c). A set volume (2 ml) of the 50% glycerol solution is sprayed, after which the samplers are removed and re-weighed, recording the value next to the correct position of each (1–4) on a data sheet. The difference between the initial weight and the weight after spraying is calculated. This process is repeated until the weight difference between each cover slip is ≤ 10% of the average of the four. Table 1 summarises each step in the treatment process for both sprayers with the time taken to complete.

The next calibration step is to confirm what volume should be pipetted into the delivery vial to deposit the required weight of spray solution onto the target surface. With the PT, most of the contents of the delivery vial are not delivered to the target surface on the spray table. Therefore, it is necessary to measure how much is actually deposited onto the target surface and then to adjust the volume of each insecticide solution needed in the delivery vial to achieve the desired spray weight [9]. The LITE method for measuring the amount of insecticide solution reaching the test surface is to spray a Petri dish containing glycerol with water; the glycerol will reduce evaporative loss of the water. The Petri dish is weighed before and after spraying to calculate the delivered spray weight. The volume aliquoted into the spray vial is adjusted until the appropriate spray deposit in weight per unit area is achieved for that spray solution (within ± 5%).

During the tests performed for this study, however, the volume deposited onto the surface was measured via fluorimetry. The fluorescent tracer is soluble, providing

Table 1 Comparative assessment of the time taken to achieve various goals

Activity		Time taken (minutes)			
		Potter Tower best case Calibration	Potter Tower worst case Calibration	Track Sprayer check calibration	Track Sprayer full Calibration
Set-up for calibration stage 1		10	10	10	10
PT calibration	Calibration stage 1: centralisation of the Potter Tower (samplers < 10% of the mean weight)	20	90	N/A	N/A
	Calibration stage 2: volume required for the desired spray weight	20	20	N/A	N/A
TS calibration	Calibration TS—3 reps carried out at 0.45 m/s for the full calibration 15 min to check settings	N/A	N/A	15	90
Time to spray		20	20	10	10
Time taken for fluorometric analysis		60	60	60	60
Time to clean		60	60	75	75
Total		190	260	170	245

Note that the PT requires calibration every time it is used whereas the TS only once every 6 months. The PT calibration time is extremely variable, and this is reflected with best- and worst-case examples

a direct volumetric measure of deposit which is not impacted by evaporative losses. The amount deposited on the spray table was measured using a 9-cm-diameter Petri dish as a collection surface; 1.7 ml of solution was sprayed, and the volume collected in the Petri dish was an average of 0.191 ± 14 ml, which translates to 30 ml/m^2, and correlates well with previous spray rate measurements. This volume represents 11.2% of the spray solution pipetted into the delivery vial, meaning that 88.8% of applied spray solution is not deposited onto the spray target. The main route of loss of spray droplets is onto the walls of the tower, with the rest likely exhausted from the bottom of the PT.

Calibration of the PT must be repeated each time it is used: the weight of deposit should be confirmed for each spray solution and the centralisation repeated before each spray session and each time the tower is cleaned, for example in between spraying different solutions. The time taken, particularly for the first centralisation step, varies considerably; during this study the time taken ranged from 50 min to 2 h 10 min. The total time to calibrate, spray test surfaces and clean the PT during this study ranged from 3 h 10 min to 4 h 20 min.

Track sprayer calibration

The Horizontal TS cabinet contains a spray table (79 × 229 cm) enclosed within a Perspex cabinet with an opening for user accessibility that is covered with a set of doors (Fig. 2). An SOP for the operation of the TS is available online at https://innovationtoimpact.org/resources/ and as Additional file 2. The spray track working length is 160 cm, and the spray table is 46.2 cm below the nozzle. In this study, deposition samplers rested on Petri dishes to give a surface height of 1.2 cm, meaning that the spray surface was 45 cm below the nozzle. This is the same nozzle distance advised for the application by backpack sprayers used for IRS in the field. The nozzle traverses the entire length of the spray track. The spray zone, however, was the central 1 m of the track; 30 cm at each end is not used because of the edge effects at the start up and shutdown of spray nozzles. The spray swath width is 70 cm, but only the central 50 cm is used because of tapering of deposits at the edge of the spray pattern. The total sprayed surface area in one run of the TS is 1.2 m^2, and the operational zone for spray target placement is 0.7 m^2, meaning that 58% of the applied volume deposits onto the targets.

The track is powered by a stepper motor governed by a control unit that offers calibrated speeds between 0.2 and 0.75 m/s with separations of 0.05 m/s. The travelling speed was set to 0.45 m/s for the experiments described here, which corresponds to 1 m sprayed every 2.2 s as per WHO guidelines for application of IRS treatment to walls [1].

The input parameters for the TS are speed, nozzle type (FF 80 02 in this model of the TS, which is the same nozzle used in the backpack sprayers during IRS operations), distance between nozzle and spray surface, and pressure. The settings to achieve the target application rate of 30 ml/m^2 were a speed setting of 0.45 m/s with a Flat fan 80 02 (spray angle and orifice size respectively) at a distance of 45 cm from the table surface. For all tests performed for this study, the tank was pressurised to 60 pounds per square inch (psi); the

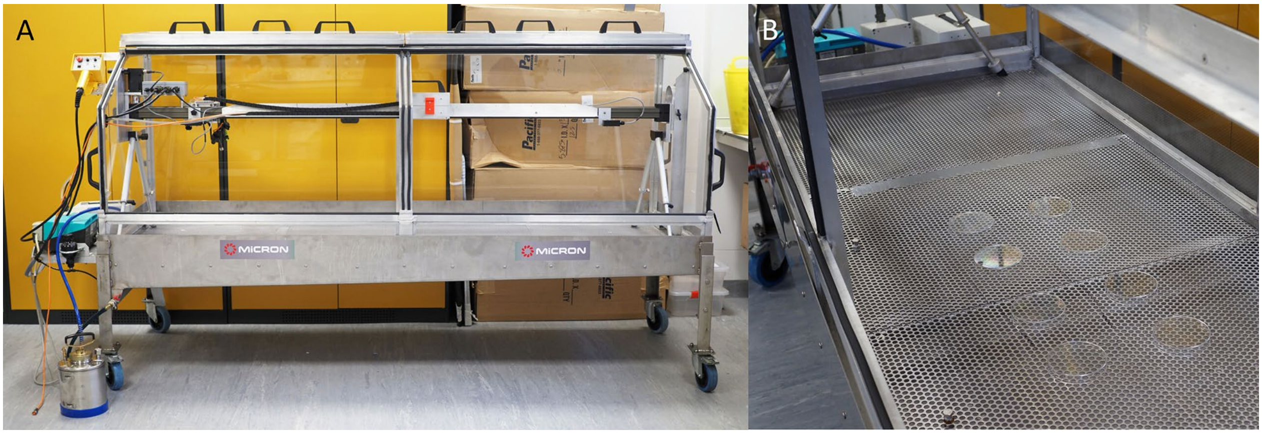

Fig. 2 The Horizontal Track Sprayer developed by Micron Sprayers. The Track Sprayer is shown empty (**A**) and with eight samplers arranged on the track for calibration (**B**)

control flow valve, however, maintains the pressure at the nozzle at 20 psi.

The track sprayer requires an in-depth periodic (every 12 months or whenever any parameter changes) calibration of the following:

Swath area: To ensure no deviation of the effective swath width (0.7 m), defined as the length under the track where spray solution is deposited evenly, water sensitive papers (Spraying System, Wheaton, Ill., 26 × 76 mm) are used that irreversibly change from yellow to blue on contact with a liquid. This test aims to identify failures due to nozzle wear; for example, over- or under-spraying on one side of the swath may be caused by wear on one side of the nozzle or a fault in manufacture. The deposition characteristics are evaluated using 10 individual sensitive papers placed width ways across the spray track, and the spray application is replicated three times. The TS passes this test if the sensitive papers are uniformly blue across the central (0.5 m) swath.

Track speed: To confirm the speed stipulated on the track controller, a known length of the track is marked, and the time for the nozzle to traverse that length is timed to enable a calculation of speed in metres/second (m/s). An alternative method is to measure the distance travelled by the belt on one revolution of the stepper motor drive and to time the number of revolutions per second.

Acute volume check: The volume deposited per unit area should be assessed via fluorimetry using the appropriate settings. There is generally a linear response between light intensity and tracer concentration when samples are not saturated, thus allowing a calculation of volume from calibration standards. If the dosage applied to the samples is incorrect, settings must be modified accordingly. The speed of the TS is the primary parameter to adjust: it can be increased and decreased to reduce and increase the volume applied, respectively.

The set up for the TS is measuring out the fluorescent tracer and the insecticide into the tank, closing the tank and pressurising to 60 pounds per square inch (psi). Full calibration of the TS takes 1 h 30 min, and this should be repeated every 6 months. Otherwise, only a routine check of sprayer settings is required, which takes 15 min. Thus, the total time to calibrate, spray and clean the TS is either 2 h 50 min or 4 h 5 min (Table 1), slightly quicker than the PT and a lot more predictable.

Sprayer cleaning

Cleaning of PT: Water is added to the atomiser reservoir and sprayed through three times. The nozzle is removed and cleaned with distilled water flushing through the feeding tube. The inside of the PT is wiped with Decon 90 on absorbent paper towel; this is repeated three times. Distilled water is then sprayed through the PT.

Time taken actively cleaning the Potter Tower: 1 h

Cleaning the TS: The exterior of the TS and associated electronic parts require minimal cleaning. All spray tubes are purged until clear; the tank is depressurised and any remaining insecticide discarded. The tank is refilled a 5% solution of Decon 90 in water, re-pressurised and rinsed through to decontaminate followed by a rinse with water alone.

Time taken to clean track sprayer tubing: 1 h 15 min every time a distinct compound is used

To clean the Perspex chamber the cabinet is first washed down with water and drained. Then, 5% Decon 90 is sprayed over the surface and left for 60 min to decontaminate, and the cabinet is wiped down. This is followed by

two more water rinses, and lastly the cabinet is sprayed with 70% ethanol, which is left to evaporate.

Time taken to clean track sprayer chamber: 1 h 15 min

Time taken includes a 60 min waiting period for Decon decontamination at the end of a treatment period.

Sprayer testing for uniformity of deposits

Fluorimetry

A series of tests were performed to evaluate the uniformity of spray deposits across an individual sprayed surface and between different sprayed surfaces (within treatment and between treatment variability). This was initially conducted as a direct volumetric assessment via fluorimetry to check the calibration of both spray systems. Both the PT and TS were calibrated to apply 30 ml/m^2 (standard operating procedures (SOPs) for calibration and use of the Potter Tower and Track Sprayer are available online at https://innovationtoimpact.org/resources/ and as Additional files 1 and 2). The sprayed surfaces were glass microscope cover slips (4.82 cm^2). Whilst the size of surfaces used in the laboratory testing of IRS treatment applications are typically much larger than a coverslip, the small surface area sprayed by the PT meant that the use of coverslips was the only practical option to assess within treatment variability. The same deposition samplers were also utilised in the TS to ensure comparability. Data analysis examined the average of the spray deposits and the variability of deposits, looking at both the within-treatment variability and between-treatment variability.

The fluorescent tracer fluorescein (Honeywell, Fluka, NC, USA, purity 98.5–100.5%) was used as a 0.01% *w*/*v* solution in water. This was an appropriate concentration to provide a linear response of the raw fluorescent units (RFU) to volume, considering the volumes expected in the rinsate used to wash the spray solution from the deposition samplers. The filters on the fluorimeter were set with excitation at 490 nm and emission at 515 nm. To analyse samples of unknown concentration, a set of known concentrations was first generated from a 0.01% stock solution of fluorescein (0, 5, 10, 20 and 30 µl of stock solution in 10 ml deionised water) and fluorescence intensity plotted by concentration. The slope of the line linking RFU and volume was calculated using a simple linear regression $Y = \mathrm{mX} + \mathrm{b}$, where Y is the response (dependent) variable, X is the predictor (independent) variable, m is the estimated slope, and b is the estimated intercept. The RFU for the blank was subtracted and y (RFU) multiplied by the slope m to determine X (the RFUs of the wash-off solutions of unknown samples). The unknown samples were collected in pre-labelled plastic bags, washed in 10 ml of water with 0.05% (or 50 mg/l) of Tween 20 (Merk, NJ, USA) and agitated by hand. Then, an aliquot of the sample was added to a cuvette (3–5 ml), and the fluorescence intensity of each sample was measured using a Trilogy Fluorometer (Turner Designs, San Jose, CA).

Deposition of active ingredient (AI)

A further set of tests were run alongside fluorimetry to check that the AI was delivered appropriately through the two spray systems. Three IRS products were used: Actellic® 300CS applied at 1 g AI/m^2 (AI pirimiphos-methyl; Syngenta, USA), K-Othrine® WG250 applied at 25 mg/m^2 (AI deltamethrin; Bayer, USA) and Suspend PolyZone applied at 25 mg/m^2 (AI deltamethrin; Bayer, USA). These tests assessed deposit volume (ml) via fluorimetry and concentration of AI via HPLC (mg).

HPLC analysis

The HPLC analysis measured the concentration of AI sprayed on the deposition samplers and the concentration in the spray solution. The HPLC analysis was performed using a Dionex UltiMate 3000 comprising an autosampler, quaternary pump and variable wavelength detector. Chromeleon 7.2 SR4 software was used for peak analysis. All AIs were analysed using a 250 × 4.6 mm Thermo Scientific Hypersil Gold C18 column with a particle size of 5 µm. Instrument methods for each AI are outlined in Table 2.

Cover slip extraction method

After spraying, coverslips were placed into individual 50-ml Falcon tubes and broken down. Five (5) ml of extraction solution (100 ug/ml dicyclohexyl phthalate [DCP] in acetone) was added so that the broken coverslips were fully submerged. The samples were sonicated for 30 min at ambient temperature to extract the insecticide from the coverslips. Then, 1 ml of the sonicated solution was transferred to a 10-ml glass tube and evaporated under compressed air at 60 ℃, leaving the insecticide residue on the walls of the glass tube. The evaporated

Table 2 HPLC instrument methods used for the active ingredients analysis

	Active ingredient	
	Pirimiphos-methyl	Deltamethrin
Injection volume	20 µl	20 µl
Wavelength	232 nm	226 nm
Run time	30 min	30 min
Mobile phase	70% Acetonitrile: 30% water	70% Acetonitrile: 30% water
Flow rate	1 ml/min	1 ml/min

samples were resuspended in 1 ml of acetonitrile by vortexing at 3000 rpm for 1 min before transferring to a 1.5-ml Eppendorf tube and centrifuging at 13,000 rpm for 20 min. Then, 100 µl of the cleaned samples was transferred to HPLC vials ready for injection.

Spray solution extraction method

Samples of the spray solution (IRS product diluted in water) were collected from the tank or the nozzle at the time points outlined. In a 10-ml glass tube, 100 µl of the formulation was diluted in 4.9 ml deionised water and vortexed. Then, 500 µl was transferred to another glass tube and evaporated under compressed air at 60 ℃. Following this, 2 ml of the extraction solution was added, and the samples were sonicated for 30 min. One (1) ml of the sonicated solution was transferred to a 10-ml glass tube and evaporated again under the same conditions. The evaporated samples were resuspended in 1 ml acetonitrile by vortexing at 3000 rpm for 1 min before being transferred to a 1.5-ml Eppendorf tube and centrifuging at 13,000 rpm for 20 min. Finally, 100 µl of the cleaned sample was transferred to HPLC vials ready for analysis.

Residual efficacy testing using WHO cone bioassays

WHO testing guidelines define residual efficacy of an IRS insecticide as the length of time after spraying that surface insecticide kills ≥ 80% of mosquitoes within 24 h of a 30-min exposure in a WHO cone test [9]. This is one characteristic of a new IRS formulation that should be demonstrated to obtain listing by the WHO vector control prequalification team. Following the calibration via fluorimetry, Actellic® 300CS was applied at the recommended application rate (1 g AI/m^2) and a quarter of this application rate (0.25 g AI/m^2) for subsequent assessment of residual efficacy via WHO cone bioassays. Both sprayers were used to apply Actellic® 300CS to the glazed surface of a plain ceramic tile using untreated glazed tiles as a negative control. Despite eight attempts to calibrate the PT, we were not able to get the weight of individual coverslips to ≤ 10% of the mean weight on this occasion. Therefore, due to a limitation on time to make the treatment applications, the tiles were sprayed without meeting this calibration criterion. In contrast, the TS maintained calibration and could be used immediately for treatment applications.

The residual performance was monitored by measuring mortality in a WHO cone test at 24 h and then at 1, 2, 3, 6, 7 and 8 months post-application of the IRS formulation to four treated surfaces [9]. Ten (10) adult female mosquitoes, 3–5 days old, mated but not blood-fed, of the susceptible Kisumu strain of *Anopheles gambiae* sensu stricto [12, 13] were exposed in WHO cones for 30 min at each time point. Knockdown (KD) was scored 60 min after exposure, and then mosquitoes were held for 24 h at 27 ± 2 °C and 70 ± 10% relative humidity before scoring mortality as reported in Table 3.

Results

Uniformity of the deposited volume

Both the PT and TS were calibrated to deposit 30 ml/m^2; on average, both sprayers deposited 31 ml/m^2. The difference between the two sprayers was in the variability in the deposits (Tables 4 and 5). Between treatments the PT had a standard deviation of 11.2 vs. a standard deviation of 3.1 for the TS. Within treatments the standard deviations were ≤ 14.7 for the PT and ≤ 4.1 for the TS.

If a calibration metric of ≤ 10% deposition deviation from the mean of all samplers is applied as a metric of pass or fail, the PT immediately fell out of calibration for uniformity of deposits. However, in all but three instances, the TS passed this calibration metric (Table 4[illegible]

Deposition of active ingredient (AI)

HPLC analysis

The HPLC analysis (January 2021) was run alongside the fluorimetry. The fluorimetry showed that the volume of liquid deposited on the surface was as expected, but HPLC analysis showed very different numbers for the AI.

The deposition volume measured via fluorimetry for Actellic 300 CS, a microencapsulated suspension, and K-Othrine WG250, a wettable granule formulation, showed that the calibrated volume of formulated product made up as a suspension in water (30 ml/m^2) was accurately deposited onto the surface.

Table 3 Spray uniformity for the Potter Tower and the Track Sprayer

	Potter Tower				Track Sprayer			
	R1	R2	R3	Average	R1	R2	R3	Average
Application (ml/m^2)	27.5	30.2	32.2	30.8	29.1	32.2	33.1	31.5
StDev	10.6	10.3	14.7	11.2	4.1	2.3	1.1	3.1
CV	36.5	38.4	34.1	38.7	14.1	7.1	3.3	9.8

Spray uniformity is calculated as a total for uniformity and uniformity across single treatment tiles. Data presented as average ml/m^2 and standard deviation

Table 4 Within-treatment variability in the Potter Tower and Track Sprayer

Potter Tower				Track Sprayer			
Average	Measured	Difference	Deviation (%)	Average	Measured	Difference	Deviation (%)
27.5	14.4	13.1	47.6	29.1	33.1	− 4.0	− 13.7
	23.6	3.9	14.4		26.2	2.9	9.8
	34.6	− 7.1	− 25.9		24.9	4.2	14.4
	37.3	− 9.8	− 35.8		32.3	− 3.2	− 11.1
30.2	18.4	11.8	43.1	32.2	34.3	− 2.1	− 6.5
	23.6	6.6	23.9		34.2	− 2.0	− 6.2
	33.3	− 3.1	− 11.4		30.7	1.5	4.8
	45.3	− 15.1	− 55.0		29.7	2.5	7.7
32.2	31.7	0.5	1.9	33.1	34.4	− 1.3	− 3.9
	52.9	− 20.7	− 75.3		33.3	− 0.2	− 0.7
	24.5	7.7	27.8		33.2	− 0.1	− 0.4
	19.5	12.7	46.0		31.6	1.5	4.6

The deviation from the mean (< 10%) is calculated as a metric of calibration for the Potter Tower and was assigned to the raw data for both sprayers

Table 5 Overview of the differences between the Potter Spray Tower and Micron Horizontal Track Sprayer for calibration, throughput and cost per tile sprayed

		Potter Spray Tower		Micron Horizontal Track Sprayer
		Best case	Worst case	
Calibration	Frequency of calibration	Every time		Every change in configuration or 6 months
	Total time for calibration and cleaning (hours)	3.2	4.3	4.1
Throughput: Assumption: 1 week of spraying (5 days, 8 h per day, two people), calibration of TS once	Number of calibrations required	5		1
	Total time for calibration per week (hours)	15.8	21.7	4.1
	Total number of work hours per week (hours)	40		40
	Time left for spraying (hours)	24.2	18.3	35.9
	Number of tiles that can be sprayed per hour 2 min per spray run	30		540
	Total number of tiles that can be sprayed per week	725	550	19395
Cost	Cost of two people per week ($20 per hour per person)	$1,600.00		
	Cost per tile	$2.21	$2.91	$0.08

- The PT deposited and average of 30.2 ± 11.8 ml/m^2 of Actellic 300CS spray solution and 29.7 ± 5.0 ml/m^2 of K-Othrine WG250 spray solution
- The TS deposited an average of 28.3 ± 2.4 ml/m^2 of Actellic 300CS spray solution and 30.8 ± 3.8 ml/m^2 of K-Othrine WG250 spray solution

In contrast, the HPLC data showed that significantly more insecticide had been deposited using the PT and significantly less using the TS. The desired application rate of Actellic 300CS on the surface was 1 g AI/m^2, and the desired concentration for K-Othrine WG250 was 25 mg AI/m^2.

- The PT deposited an average of 2137 ± 607 mg AI/m^2 for Actellic 300CS and an average of 75.5 ± 8.7 for K-Othrine WG250
- The TS deposited an average of 92.4 ± 13.9 mg AI/m^2 for Actellic and 8.4 ± 0.9 mg AI/m^2 for K-Othrine WG250

The PT deposited approximately 2×and 3×the intended dose of Actellic 300CS and K-Othrine WG250, quantified by the amount of AI recovered and measured by HPLC, respectively, whilst the TS deposited a 10th and a 3rd of the intended dose, respectively.

Tank samples (spray solution) throughout testing with K-Othrine WG250 were analysed, which flagged a potential flaw with the TS; there was a sudden drop in the concentration from the tank sample taken post spray (Fig. 3). Visual inspection of the TS showed signs of the AI sedimenting out in the lines. The suspension of diluted product in the tank can be maintained by constant agitation. The sedimentation of the formulation can be addressed by flushing out the lines after any significant stoppage in spray applications alongside regular agitation of the spray tank. At the same time, samples were taken from the stock solution of K-Othrine WG250 before and after spraying with the PT, and directly from the nozzle, which confirmed that the AI content remained constant, and no significant sedimentation was occurring (Fig. 3).

With sedimentation of the IRS formulation identified as the likely cause of the observed under spray with the TS, a follow-up set of tests were run to compare K-Othrine WG250 with another formulation of deltamethrin with more favourable suspension characteristics (Suspend PolyZone): the recommended application rate of both formulations is 25 mg AI/m^2. The TS spray tank was agitated immediately prior to pumping to target pressure and applied promptly to spray surfaces to prevent sedimentation within the delivery tubes during set up.

Measurement of the volume of spray deposited via fluorimetry from K-Othrine WP250 and Suspend PolyZone applications showed that the calibrated volume of formulated product (30 ml/m^2) was deposited on the surface.

- The PT deposited an average of 31 ± 2.8 ml/m^2 for K-Othrine WG250 and an average of 32 ± 2.3 ml/m^2 (SD 2.3) for Suspend PolyZone.
- The TS deposited an average of 31 ± 1.6 ml/m^2 for K-Othrine WG250 and 30 ± 1.9 ml/m^2 for Suspend PolyZone.

The HPLC analysis showed that the improved agitation of the TS system to stop sedimentation of AI returned the desired concentrations of deltamethrin sprayed in

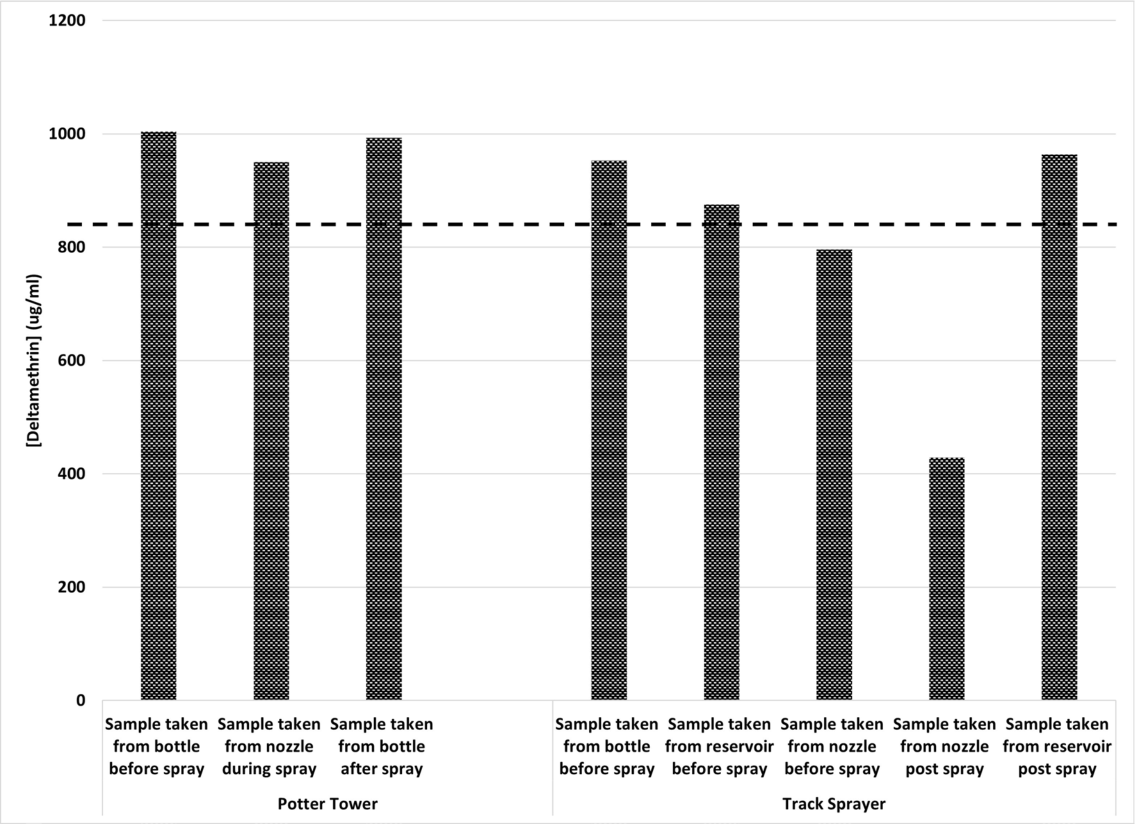

Fig. 3 HPLC analysis of active ingredient of tank samples throughout the spraying process using the Potter Tower and Track Sprayer. Dotted line represents the expected concentration of 833.3 μg AI/ml

the two formulations. The PT always deposited approximately 20% more than the target application rate for both formulations.

- The PT deposited 31 ± 3.8 mg AI/m^2 for K-Othrine WG250 and 29 ± 3.9 mg AI/m^2 for Suspend PolyZone
- The TS deposited 25 ± 2.5 mg AI/m^2 for K-Othrine WG250 and 25 ± 1.9 mg AI/m^2 for Suspend PolyZone

Both formulations were thus still over-applied when using the PT, but not to the same degree as seen previously. Using a formulation with improved suspension qualities showed some improvement, meaning the over-application could result from the AI partitioning during the descent of droplets through the tower. Ultimately, however, the reason for the over-application is still unknown. What can be seen is that the deviation in the deposition of AI from the target dose exceeds the uniformity metric of 10% when using the PT but not when using the TS.

WHO cone bioassays

The bio-efficacy data from the first trials showed that Actellic 300CS applied using the PT and TS at the recommended label rate and one-quarter of this rate delivered a deposit of pirimiphos-methyl which remained bioactive (≥ 80% mortality in mosquitoes exposed to treated surfaces in a WHO cone test) for at least 3 months (Fig. 4). At one quarter label rate (Fig. 4a), bioefficacy declined after 3 months regardless of how the IRS was applied, though tiles treated with the TS remained bioactive for longer: the TS-sprayed tiles gave 82% mortality and the PT-sprayed tiles 62% mortality at 6 months post-application. Tiles sprayed with the full label rate (Fig. 4b) using the PT showed a similar reduction in bioefficacy and failed (< 80% mortality) at 6 months, but those treated with the label rate using the TS remained bioactive until month 7 and only failed at month 8.

The second round of testing applied Actellic 300CS and K-Othrine WG250 at the recommended application rate. The AI concentration on all surfaces was measured by HPLC analysis. K-Othrine WG250 remained active, killing 100% of mosquitoes in a cone test for 3 months (Fig. 5). Actellic 300CS had reduced efficacy at 2 months when applied using both the PT and TS; at 3 months mortality rebounded up to 100% with the PT-sprayed surfaces but further declined with the TS-sprayed surfaces. This matches other observations that the TS routinely shows logical progressions, whereas the data associated with the PT tend to be more erratic, possibly because of the more variable spray deposition profiles. HPLC analysis showed that the PT deposited significantly higher rates of AI compared to the TS, which is at odds with the bioassay results where mortality was not strikingly different between the TS- and PT-treated tiles.

Discussion

To accurately compare the bioefficacy of new IRS formulations, it is essential to be able to apply them onto representative surfaces at defined and precise application rates and with even deposits across each surface. In addition, when evaluating several prototype IRS formulations, it is important to have an application method that allows the treatment of sufficient replicate surfaces of multiple treatments in a manageable length of time. This study set out to compare the current accepted method for treating surfaces in a laboratory setting, the Potter Spray Tower (PT) and the newly commissioned Horizontal

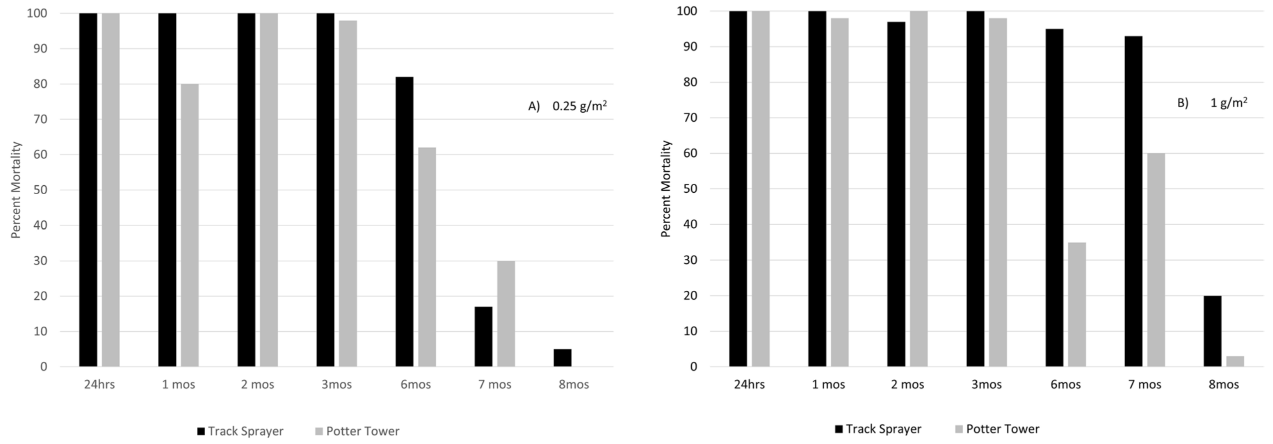

Fig. 4 Tarsal contact assay results showing mortality over time post treatment of tiles. Percentage mortality in susceptible Kisumu mosquitoes 24 h after exposure to tiles treated with pirimiphos-methyl IRS in a tarsal contact bioassay at **a** a quarter or **b** 100% of the recommended dose using a Track Sprayer or Potter Tower

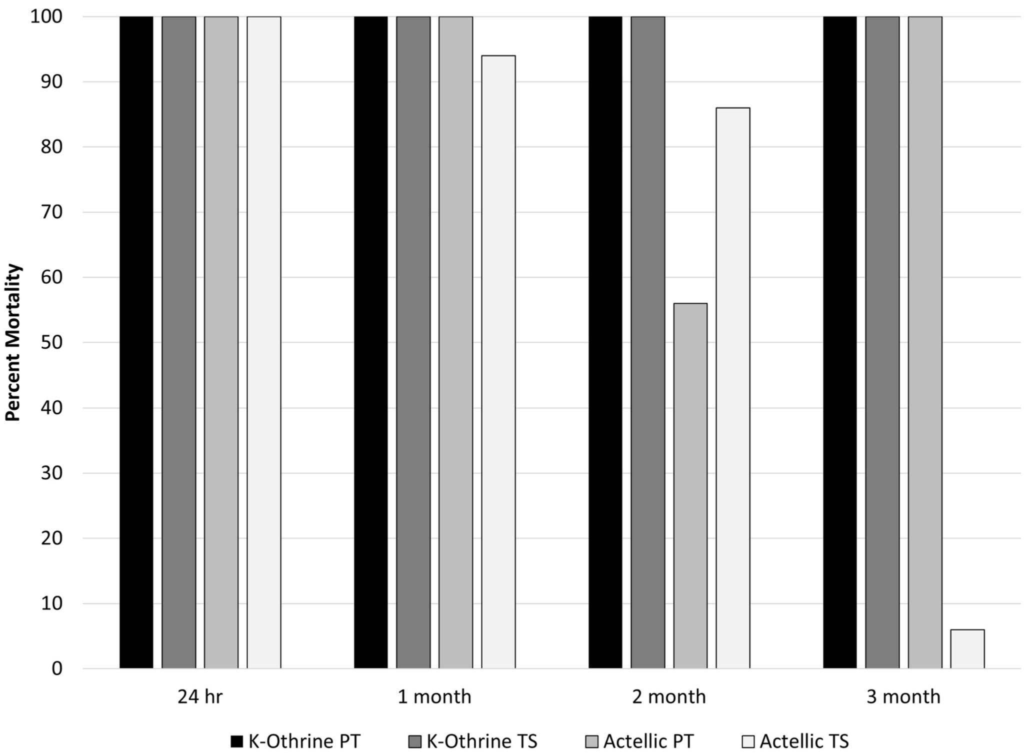

Fig. 5 Second tarsal contact bioassay with the full dose of K-Othrine and Actellic. Percentage mortality 24 h after exposure to tiles treated with Actellic 300CS or K-Othrine WG250 at the recommended application rate using either the Potter Tower or Track Sprayer

Track Sprayer from Micron Sprayers Ltd. (TS), in terms of ease and speed of operation and other practical considerations, accuracy and precision of spray deposit, and residual efficacy of sprayed deposits against mosquitoes. Table 5 shows a summary of the results of comparison between the two methods.

In terms of treatment time, the PT and TS take a similar time per application or run. However, one run of the PT only treats one surface whereas 18 surfaces of the same size (15 × 15 cm) can be treated with one run of the TS. In addition, the PT only applies spray solution onto a circle in the centre of a treated tile, which is suitable for a WHO cone test, but the TS treats the whole tile, which gives added versatility.

Given the cost of new insecticides and formulations, particularly those which are in development and may only be available in small amounts for laboratory evaluation, is it important that they are used efficiently. In terms of spray accountability, the percentage of spray landing on the target was calculated for each piece of equipment. This showed that the PT deposits < 12% of the applied dose to the target surface, which is wasteful of insecticides compared to the TS, which delivers 58% to the treated surfaces. With the PT, most of the content of the delivery vial coats the side of the spray column or is exhausted from the bottom of the tower as opposed to the target surface [9]. It is also acknowledged that differences in temperature and humidity affect the deposit on a target situated on the spray table because of the evaporation of the water carrier [10, 14]. On the other hand, with careful calculation of the amount of product required, the PT has minimal wastage compared to the TS, which generates at least 70 ml of waste product in the lines. More than 40 surfaces would have to be sprayed with the PT to exceed the 70 ml chemical waste in the lines of the TS. In this way, the PT may be preferable when using a particularly costly insecticide or formulation or treating only a small number of surfaces. With either method, insecticide-contaminated spray solution and the water and materials used to clean the spray equipment must be disposed of as chemical waste according to local regulations and logistics, using for example a remediation facility, biobed or evaporation tank.

In setting out to compare the accuracy and precision of spray deposits, the initial testing took a direct volume measure of spray deposits by each method and showed that both sprayers could be effectively calibrated to deposit the target dose of 30 ml/m^2. The key difference between the performance of the two systems was in the uniformity of the spray deposits; the variability of the dose was far greater for the PT, whilst the TS was consistently within the uniformity metric of ≤ 10% deviation

from the mean. The HPLC analysis, however, showed that the concentration of the AI in that volume of IRS formulation was orders of magnitude higher than the target dose for the PT and somewhat lower for the TS. The issue detected with the TS was sedimentation in the lines, which can be avoided with care. Misapplications in the PT have previously been put down to the evaporation of water from spray droplets, but the volumetric measures via fluorimetry with a soluble tracer discount that. Furthermore, stock solution checks showed that the correct concentration was being pipetted into the delivery vial. This would suggest a partitioning of the AI as the spray descends through the tower. The use of a formulation with better suspension characteristics reduced the amount over-applied, but the best result still represented a 20% over-application of treatment compared to the target.

While is it important that spray equipment used to treat tiles for bioefficacy testing and product development delivers the correct quantity of active ingredient (AI), usually an insecticide, it is also important that this insecticide is delivered in such a way that it is active against the mosquito target and that this activity lasts for the intended life of an IRS spray, typically 6 months. The initial tarsal contact bioassays on tiles in this study treated tiles with Actellic 300CS. The concentration of AI was not measured after application, but the tiles treated using the TS exceeded the 80% mortality threshold in a cone bioassay for 6 months for the quarter dose and 7 months for the full dose. Surfaces treated using the PT fell below this threshold sooner. The second round of bioefficacy testing showed that with the PT both AIs were applied at higher concentrations than intended to the target surface. K-Othrine WG250 exceeded the 80% threshold for the 3 months when applied by both sprayers. Actellic 300CS deposits from the PT gave inconsistent results, even with 2 × the intended dose, with a failure to kill 80% at 2 months, followed by a rebound at 3 months. The TS, which applied only a 10th of the target dose, started to lose control in a stepwise fashion returning < 80% mortality by the third month.

A further advantage of using the TS is that it simulates actual IRS practice as it uses the same nozzle, a ceramic C8002E fan nozzle and a control flow valve, but in a sealed chamber with controlled speed and consistent distance from the target. The spray deposit and subsequent transfer to the biological target can be more representative and consistent between replicates.

Conclusions

We have demonstrated that the Horizontal Track Sprayer from Micron Sprayers Ltd. (TS) can accurately dose testing surfaces with IRS products more uniformly than the current state of the art Potter Spray Tower (PT), is quicker and simpler to operate, has a higher throughput and reduces costs per treated surface.

Laboratory testing will always be a proxy for the predicted bio-efficacy or residual efficacy of an IRS formulation, but if spray applications can be applied using a similar method to that used to treat walls in IRS campaigns, this may improve the ability of laboratory tests to predict real-world performance. The TS utilises all the main components of the compression sprayers used to apply IRS. This is in contrast to the very different application technology of the PT, which uses a misting nozzle (mists are ultra-fine sprays < 100 µm) and is less representative of IRS in the field, which uses a fine to medium spray (240 µm). The new TS is more representative of IRS in the field and provides several significant additional benefits over the PT.

For both practical reasons of reliability and resource requirement and the fact that it is more representative of real-world spray conditions, we would recommend a paradigm shift away from the PT to the TS.

Abbreviations

AI	Active ingredient/s
CFV	Controlled flow valve
HPLC	High performance liquid chromatography
IRS	Indoor residual spray
IVCC	Innovative vector control consortium
LITE	Liverpool insect testing establishment
LSTM	Liverpool school of tropical medicine
PSI	Pounds per square inch
PT	Potter spray tower
SOP	Standard operating procedure
TS	Track Sprayer (Micron Horizontal Track Sprayer)
WHO	World Health Organization

Supplementary Information

The online version contains supplementary material available at https://doi.org/10.1186/s13071-024-06168-x.

Additional file 1. Standard Operating Procedure (SOP): Application of compounds to surfaces using the Potter Tower, provided by the Liverpool Insect Testing Establishment (LITE) at the Liverpool School of Tropical Medicine (LSTM).

Additional file 2. Standard Operating Procedure (SOP): Operation and maintenance of the Track Sprayer, provided by the Liverpool Insect Testing Establishment (LITE) at the Liverpool School of Tropical Medicine (LSTM).

Acknowledgements

This project was initiated by Dave Malone who at the time was Director of Technical Development at IVCC and Zikmund Bartonicek and Daniel McDermott, former MSc students at the Liverpool School of Tropical Medicine. The authors would like to thank members of Liverpool Insect Testing Establishment (LITE) team at LSTM for rearing all mosquitoes. We would also like to thank the Micron Sprayers Ltd, manufacturers of specialist spraying equipment, for designing the track sprayer and for their continued support.

Author contributions
JB, JC, RSL, DM and JC designed the study. GP and AM performed the experimental work with the Track Sprayer and Potter Tower, and conducted bioassays to produce the data for Figs. 4 and 5. KJW performed all HPLC analysis and produced the data in Fig. 3. All authors were involved in analysing and interpreting results. JB wrote the main manuscript text and prepared the figures, with significant input from RSL and DN. All authors reviewed the manuscript.

Funding
This study was funded by the Innovative Vector Control Consortium (IVCC), UK, through BMGF (INV-007509) and USAID (AID-OAA-A-16-00083) funding grants.

Availability of data and materials
The data supporting the findings of the study must be available within the article and/or its supplementary materials, or deposited in a publicly available database.

Declarations

Ethics approval and consent to participate
Not applicable.

Consent for publication
All of the material is owned by the authors and no permissions are required.

Competing interests
One of the authors, John Clayton, is an employee of Micron Sprayers Ltd., who developed the Track Sprayer, and has been involved in the validation of the equipment and the preparation of this manuscript.

Author details
[1]Bonds Consulting Group LLC, 3900 Wasp Street, Panama City Beach, FL 32408, USA. [2]Department of Vector Biology, Liverpool School of Tropical Medicine, Liverpool L3 5QA, UK. [3]Innovation to Impact, Liverpool School of Tropical Medicine, Pembroke Place, Liverpool L3 5QA, UK. [4]Innovative Vector Control Consortium (IVCC), Liverpool School of Tropical Medicine, Liverpool L3 5QA, UK. [5]Micron Sprayers Ltd, Bromyard Industrial Estate, Bromyard HR7 4HS, UK. [6]Bill & Melinda Gates Foundation, 500 5th Ave N, Seattle, WA 98109, USA.

Received: 8 August 2023 Accepted: 27 January 2024
Published online: 16 February 2024

References

1. World Health Organization. Indoor residual spraying: an operational manual for indoor residual spraying (IRS) for malaria transmission control and elimination. World Health Organization. 2015. https://www.who.int/publications/i/item/9789241508940. Accessed 21 Oct 2023.
2. Roll Back Malaria Partnership. Global Malaria Action Plan (GMAP). 2008. https://www.unhcr.org/media/global-malaria-action-plan-gmap. Accessed 21 Oct 2023.
3. World Health Organization. Prequalified Vector Control Products, WHO-Prequalification of Medical Products (IVDs, Medicines, Vaccines and Immunization Devices, Vector Control). https://extranet.who.int/pqweb/vector-control-products/prequalified-product-list. Accessed 21 Oct 2023.
4. Ranson H, Lissenden N. Insecticide resistance in African *Anopheles* mosquitoes: a worsening situation that needs urgent action to maintain malaria control. Trends Parasitol. 2016;32:187–96.
5. Lees R, Praulins G, Davies R, Brown F, Parsons P, White A, et al. A testing cascade to identify repurposed insecticides for next-generation vector control tools: screening a panel of chemistries with novel modes of action against a malaria vector. Gates Open Res. 2019;3:1464.
6. Lees RS, Ambrose P, Williams J, Morgan J, Praulins G, Ingham VA, et al. Tenebenal: a meta-diamide with potential for use as a novel mode of action insecticide for public health. Malar J. 2020;19:1–17.
7. Lees RS, Praulins G, Lissenden N, South A, Carson J, Brown F, et al. The residual efficacy of SumiShield™ 50WG and K-Othrine® WG250 IRS formulations applied to different building materials against *Anopheles* and *Aedes* mosquitoes. Insects. 2022;13:112.
8. Hemingway J, Beaty BJ, Rowland M, Scott TW, Sharp BL. The innovative vector control consortium: improved control of mosquito-borne diseases. Trends Parasitol. 2006;22:308–12.
9. World Health Organization. Guidelines for testing mosquito adulticides for indoor residual spraying and treatment of mosquito nets. https://www.who.int/publications/i/item/WHO-CDS-NTD-WHOPES-GCDPP-2006.3. Accessed 21 Oct 2023.
10. Potter C. An improved laboratory apparatus for applying direct sprays and surface films, with data on the electrostatic charge on atomized spray fluids. Ann Appl Biol. 1952;39:1–28.
11. Snetselaar J, Lees RS, Foster GM, Walker KJ, Manunda BJ, Malone DJ, et al. Enhancing the quality of spray application in IRS: evaluation of the micron track sprayer. Insects. 2022;13:523.
12. Williams J, Flood L, Praulins G, Ingham VA, Morgan J, Lees RS, et al. Characterisation of *Anopheles* strains used for laboratory screening of new vector control products. Parasites Vectors. 2019;12:522.
13. Lees RS, Armistead JS, Azizi S, Constant E, Fornadel C, Gimnig JE, et al. Strain characterisation for measuring bioefficacy of ITNs treated with two active ingredients (Dual-AI ITNs): developing a robust protocol by building consensus. Insects. 2022;13:434.
14. Garcerá C, Moltó E, Chueca P. Factors influencing the efficacy of two organophosphate insecticides in controlling California red scale, *Aonidiella aurantii* (Maskell). A basis for reducing spray application volume in Mediterranean conditions. Pest Manag Sci. 2014;70:28–38.

Publisher's Note

Springer Nature remains neutral with regard to jurisdictional claims in published maps and institutional affiliations.
